# Sustainable Tea Cultivation with a Rhizobacterial Consortium: A Microbiome-Driven Alternative to Chemical Fertilizers

**DOI:** 10.3390/microorganisms13081715

**Published:** 2025-07-22

**Authors:** Silpi Sorongpong, Sourav Debnath, Praveen Rahi, Biswajit Bera, Piyush Pandey

**Affiliations:** 1 Soil and Environmental Microbiology Laboratory, Department of Microbiology, Assam University, Silchar 788011, India; sorongpongsilpi@gmail.com (S.S.); souravdebnath020@gmail.com (S.D.); 2 National Centre for Cell Science, Pune 411007, India; praveen_rahi22@yahoo.co.in; 3 Institut Pasteur du Cambodge, 5 Preah Monivong Blvd., St. 93, Phnom Penh 120210, Cambodia; 4 Tea Board of India, Kolkata 700001, India; dr1teaboard@gmail.com

**Keywords:** *Camellia sinensis*, PGP attributes, phenol, catalase (CAT), glutathione, superoxide dimutase (SOD), bacterial community

## Abstract

The excessive use of chemical fertilizers in tea cultivation threatens soil health, environmental sustainability, and long-term crop productivity. This study explores the application of plant growth-promoting bacteria (PGPB) as an eco-friendly alternative to conventional fertilizers. A bacterial consortium was developed using selected rhizobacterial isolates—*Lysinibacillus fusiformis*, five strains of *Serratia marcescens*, and two *Bacillus* spp.—based on their phosphate and zinc solubilization abilities and production of ACC deaminase, indole-3-acetic acid, and siderophores. The consortium was tested in both pot and field conditions using two tea clones, S3A3 and TS491, and compared with a chemical fertilizer treatment. Plants treated with the consortium showed enhanced growth, biomass, and antioxidant activity. The total phenolic contents increased to 1643.6 mg GAE/mL (S3A3) and 1646.93 mg GAE/mL (TS491), with higher catalase (458.17–458.74 U/g/min), glutathione (34.67–42.67 µmol/gfw), and superoxide dismutase (679.85–552.28 units/gfw/s) activities. A soil metagenomic analysis revealed increased microbial diversity and the enrichment of phyla, including *Acidobacteria*, *Proteobacteria*, *Actinobacteria*, *Chloroflexi*, and *Firmicutes*. Functional gene analysis showed the increased abundance of genes for siderophore biosynthesis, glutathione and nitrogen metabolism, and indole alkaloid biosynthesis. This study recommends the potential of a PGPB consortium as a sustainable alternative to chemical fertilizers, enhancing both the tea plant performance and soil microbial health.

## 1. Introduction

Tea [*Camellia sinensis* (L.) O. Kuntze] (Theaceae) is the source of the most extensively consumed non-alcoholic beverages throughout the world and has many health benefits due to its antioxidant, antibacterial, antimutagenic, and anticancer functions [1,2]. In an effort to improve the productivity and growth of tea plants, growers have been indiscriminately using excessive chemical fertilizers and pesticides, prioritizing short-term economic gains over long-term sustainability. However, the regular use of chemical fertilizers has a negative impact on the environment, plants, and human health [3,4]. Research suggests that the use of bacteria with plant growth-promoting attributes could significantly reduce the reliance on chemical fertilizers while simultaneously improving the tea yield and quality [5]. The efficiency and effectiveness of organic amendments have always been a concern among tea growers. China, India, and Sri Lanka dominate the world’s tea production [6]. With almost 10% of global tea exports, India ranks among the top five exporters. The largest tea plantation in India is in Assam, followed by Darjeeling in West Bengal. India’s total tea export during 2023–2024 was 250.73 million kg and worth USD 776 million [7]. There are challenges associated with declining tea quality and rising tea prices, which arise due to the age of tea bushes, excessive fertilizer use, climatic change, and a change in the industry’s paradigm [8,9]. Global tea consumption is projected to have increased by approximately 43% between 2005 and 2020, with the majority of the tea production taking place in tropical and subtropical regions, such as India. As a result, several studies have been undertaken to understand how fertilizers affect the soil conditions, as well as the tea yield and quality [10].

Tea has important physiological effects due to the presence of compounds such as polyphenols, amino acids, vitamins, carbohydrates, caffeine, and purine alkaloids, all of which are known to have health benefits. Adequate nitrogen (N), phosphorus (P), and potassium (K) accessibility is indispensable for tea growth, which is often limited in agricultural soil and frequently supplemented by chemical fertilizers [11]. The demand for organic tea is increasing due to the influence of health concerns and the resultant food habits of tea consumers. As its commercial potential grows, the demand for certified organic tea (COT) is rising in the global market. The excessive use of synthetic fertilizers contributes to soil degradation, greenhouse gas emissions, and water pollution, exacerbating climate change impacts, which is reflected in the Sustainable Development Goals (SDG 13: Climate Action). Organic farming minimizes chemical inputs, supports biodiversity, and enhances soil health, aligning with SDG 2 (Zero Hunger) by promoting sustainable food production systems [12]. Additionally, sustainable tea cultivation contributes to SDG 12 (Responsible Consumption and Production) by reducing environmental footprints, and to SDG 15 (Life on Land) by preserving soil ecosystems. Transitioning towards eco-friendly practices in tea farming can mitigate the effects of climate change while ensuring long-term agricultural sustainability.

A sustainable approach to the production of organic tea involves the use of biofertilizers prepared from plant growth-promoting bacteria (PGPB), which typically reside in the rhizospheres of plants. PGPB are considered potential eco-friendly alternatives to chemical fertilizers. PGPB are known to promote plant growth through both direct and indirect mechanisms. They help plants by producing plant growth-promoting phytohormones, such as indole acetic acid (IAA); solubilizing inorganic phosphate and potassium; and producing siderophores and ACC deaminase [13]. Previous studies have found that *Bacillus* and *Serratia* spp. have significant potential for the growth promotion of plants as PGPB [1,14]. Reducing chemical inputs through the use of efficient bacterial formulations offers a sustainable approach to stabilize yields and minimize environmental impacts [15]. Several studies have previously demonstrated the effectiveness of PGPB at promoting plant growth and improving soil fertility across various crops, including tea. For instance, there are reports on the role of *Chloroflexi* sp. in phosphate solubilization, which can improve tea plant vigor [16]. Similarly, *Paraburkholderia hospita* and *Sinomonas gamaensis* improved the lateral root growth in tea due to their growth-promoting attributes [17]. There have been several reports where the plant growth, root length, and young shoot productivity significantly increased in tea plants due to PGPB treatments, as presented in the reviews [15,18], even though there have been concerns about the effectiveness of bacterial isolates in the field, and their impact on the indigenous bacterial community.

Despite several studies demonstrating the role of rhizospheric bacteria in promoting tea plant growth, few have investigated the integrated impact of bacterial consortia on both plant physiological parameters and soil metagenomic profiles under field conditions. While previous research has predominantly explored the growth-promoting impacts of rhizobacteria in *C. sinensis* [18,19], this study takes a comprehensive systems-level approach. It integrates detailed analyses of the soil physicochemical properties, functional soil enzyme assays, and high-resolution metagenomic profiling. This holistic approach elucidates the intricate interactions among microbial community structures, their functional capabilities, and key indicators of soil health. Consequently, this research significantly advances our knowledge of the microbial mechanisms crucial for promoting sustainable tea production. Furthermore, no prior studies have simultaneously evaluated the soil enzymatic dynamics, microbial functional potential, and nutrient cycling in the tea clones S3A3 and TS491.

Hence, the present study was undertaken to identify and characterize the effectiveness of the consortium in promoting the growth of tea plants. A multispecies consortium of PGPB was strategically formulated and applied under in situ field conditions to assess its efficacy at promoting the growth of *C. sinensis* in comparison to conventional chemical fertilizers. Key agronomic and physiological parameters—including the shoot length, leaf area, chlorophyll content, and enzymatic activities in both plants and rhizosphere soil—were systematically evaluated. Furthermore, a comparative metagenomic analysis was conducted to assess shifts in the microbial diversity and community structure in response to PGPB consortium versus chemical fertilizer application within the tea plantation soil ecosystem.

## 2. Materials and Methods

### 2.1. Sample Collection

Soil samples from the rhizosphere and non-rhizosphere regions of tea plants were collected from various tea estates in Assam and Darjeeling, India. The sites in Assam included the Panitola Tea Estate (27°49′52.74″ N, 95°27′55.21″ E), Dilli Tea Estate (27°16′37.13″ N, 95°36′97.99″ E), Tocklai Tea Research Institute (26°73′02.77″ N, 94°22′74.75″ E), and three estates in southern Assam: the Silcoorie Tea Estate (24°69′85.25″ N, 92°75′85.37″ E), Roskandy Tea Estate (24°67′00.83″ N, 92°69′87.32″ E), and West Jalinga Tea Estate (24°66′43.34″ N, 92°70′83.76″ E). In Darjeeling, samples were collected from Chota Old Sec, Fulbari Sec, Sungma, Turzum R1, and Turzum R2 (27.0410° N, 88.2663° E). Rhizospheric and non-rhizospheric soils, roots, and leaves were collected and stored at 4 °C.

### 2.2. Isolation of Rhizobacteria from Soil Samples

Bacteria were isolated from rhizospheric soil using the serial dilution method. One gram of soil was aseptically suspended in 9 mL of distilled water and serially diluted up to 10^6^, and a suitable dilution was plated on nutrient agar. Plates were incubated at 37 °C for 48 h. Pure cultures of the isolates were stored at 4 °C for further analysis [11].

### 2.3. Plant Growth-Promoting (PGP) Attributes of Rhizobacterial Isolates

#### 2.3.1. Phosphate Solubilization and Zinc Solubilization

The phosphate solubilization was qualitatively estimated by the plate method. The bacterial isolates were inoculated in Pikovskaya medium [20] (containing bromophenol blue) and incubated at 37 °C for 48 h. The presence of a yellow-colored halo zone around the colonies was considered a positive indication of phosphate solubilization [13]. For the quantitative estimation of phosphate solubilization, the isolates were inoculated in a National Botanical Research Institute phosphate growth medium (NBRIP, Section S3 of Supplementary Materials) (pH 7) and incubated at 150 rpm at 30 °C. After every 24 h, samples were collected and centrifuged, and the phosphate concentration was determined by the vanadomolybdate phosphoric acid yellow color method [14]. The zinc solubilization activity was determined using the method modified from Yasmin et al. [21]. Halo zone formation in Basal medium plates containing 0.2% insoluble Zinc Oxide (ZnO) confirmed the solubilization of Zn.

#### 2.3.2. Siderophore Production

The ability of isolates to produce siderophores was evaluated using the chrome azurol S (CAS) agar plate assay. Bacterial isolates (5 μL) were inoculated onto CAS agar plates and incubated at 28 ± 2 °C for 72 h. Siderophore production was indicated by a change in the medium’s color from blue to orange. Siderophores were quantified using the CAS shuttle method, and the isolates were inoculated into an iron-deficient broth medium. After every 24 h, 5 mL of culture was taken out and centrifuged at 10,000 rpm for 10 min. Culture supernatant (0.5 mL) with 0.5 mL of CAS reagent was mixed, and the absorbance was recorded at 630 nm using a UV spectrophotometer (Eppendorf AG BioSpectrometer, Hamburg, Germany). CAS agar medium and other reagents were prepared as reported by Pandey et al. [22]; the compositions are provided in the Section S3 of Supplementary Materials.

#### 2.3.3. ACC Deaminase

The ACC deaminase activity was screened using the plate method. Dworkin and Foster (DF) salt plates supplemented with 3 mM ACC were inoculated with isolates and incubated at 28 °C for 3 days to observe growth, if present. The ACC deaminase activity was quantified spectrophotometrically by measuring the α-ketobutyrate formation at 540 nm. The total protein was determined using the Bradford method. The enzyme activity was reported as nmol α-ketobutyrate produced per hour per mg of cellular protein [23].

#### 2.3.4. IAA, Ammonia, and HCN Production

The isolates were cultivated for five days at 30 °C in a shaker incubator at 150 rpm in Nutrient Broth supplemented with 2 mg/mL tryptophan. To determine the amount of IAA produced, the bacterial culture was centrifuged at 10,000 rpm for 10 min, and Salkowski reagent was added to the supernatant. The absorbance was measured at 530 nm [24].

To determine the ammonia production, the isolates were cultured in peptone broth and incubated for 48–72 h at 28 ± 2 °C. The bacterial suspension was treated with Nessler’s reagent (0.5 mL). The development of a brown to yellow color indicated ammonia production [25].

For HCN production, the isolates were grown on nutrient agar supplemented with 0.44% glycine. Whatman filter paper soaked with 2% sodium carbonate and 0.5% picric acid solution was placed over the plate and sealed. Plates were incubated at 30 °C for four days. A color change of the filter paper from yellow to orange or brown was indicative of HCN production [26].

### 2.4. Identification of Rhizobacterial Isolates

Bacterial isolates with prominent PGP attributes were identified through 16S rRNA sequence analysis. The PCR amplification of the 16S rRNA gene used the primers 27F (5′-AGAGTTTGATCCTGGCTCAG-3′) and 1492R (5′-TACGGYTACCTTGTTACGACTT-3′) with conditions of 94 °C for 3 min initial denaturation, 94 °C for 30 s denaturation, 54 °C for 1 min annealing, 72 °C for 1.5 min extension, and a final 10 min extension at 72 °C. PCR products were resolved by 1.5% agarose gel electrophoresis, purified using the QIAquick Gel Extraction Kit (Qiagen, New Delhi, India), and sequenced (Applied Biosystem 3730xl Sequencer, Thermo Fisher Scientific, Mumbai, India). Sequences were identified using BLASTn version 2.16.0+ (NCBI) and analyzed using DNA Baser (v2.9.54). Phylogenetic analysis was performed using the neighbor-joining method in MEGA 11 with the Tamura–Nei model [27].

### 2.5. Pot and Field Trial Assays with C. sinensis Plants—S3A3 and TS491 Clones

#### 2.5.1. Greenhouse Assay

Six-month-old tea clone seedlings of *C. sinensis* var. *assamica* (S3A3 and TS491, 15–20 cm tall) grown in 16 cm × 20.5 cm × 21 cm polyethylene sleeves were used for a pot experiment. Pots (15 cm × 15 cm × 30 cm) were filled with 7 kg of soil and planted with S3A3 and TS491 under greenhouse conditions maintained at 25 ± 2 °C with a relative humidity of 60–70% under natural daylight conditions. Four treatments were applied: (a) a control (no inoculation), (b) a bacterial consortium, (c) 50% fertilizer + consortium, and (d) fertilizer (only). The treatment abbreviations were systematically coded to represent the sample type, treatment condition, and time point. Each code consists of a prefix followed by a numerical identifier. The prefix indicates the sampling stage and variety. “IS” and “IT” denote Initial Samples of the S3A3 and TS491 varieties, respectively (i.e., before treatment), while “FS” and “FT” indicate Final Samples (i.e., after treatment). The numerical suffix with S (1–4) specifies the treatment: S1—control, S2—consortium, S3—50% fertilizer + consortium, and S4—fertilizer only. This coding scheme enables clear tracking and a comparison of the treatments across the time points and varieties.

Seedlings were planted with a complete randomized design and three replicates per treatment. The commercially available compound fertilizers (N:P:K) were applied at a 2:1:3 ratio (urea, diammonium phosphate, and muriate of potash) [28]. For the preparation of the bacterial consortium, the selected bacteria were cultured separately in a Nutrient Broth medium for 72 h at 28 °C and then centrifuged (10,000× *g*, 10 min), harvested, washed, and suspended in sterile saline to a final cell density of ~108 CFU mL^−1^. The bacterial consortium of 8 isolates was developed by combining the same quantity (5 mL) of each individual bacterium (OD600 ~1.0) with a total volume of 40 mL consortium per preparation. Each strain was selected based on complementary plant growth-promoting traits. The strains were tested for mutual growth using standard cross-streak culture methods on a nutrient agar medium. Isolates were considered compatible if there was no inhibition zone [29]. The consortium treatment was repeated every 15 to 30 days for all treatments except for the control and fertilizer treatments. Plants were watered as needed to maintain optimal soil moisture, avoiding both water stress and waterlogging. After six months, at an interval of three months, the tea plants were harvested and evaluated for their shoot lengths, root lengths, leaf numbers, chlorophyll contents, enzyme activities, and relative water contents. The rhizospheric soil was also collected, and the soil enzyme activity was analyzed [28]. For the analysis of the pigments (chlorophyll), enzyme activities, and leaf relative water content (LRWC), fully expanded middle leaves from each plant were used in the pot trial and field trial.

#### 2.5.2. Field Assay

Field trials with six-month-old tea clone seedlings of *C. sinensis* var. *assamica* (S3A3 and TS491, 15–20 cm tall) grown in 16 cm × 20.5 cm × 21 cm polyethylene sleeves were used to evaluate the effectiveness of the respective plant–rhizobacterial consortium interaction. The same fertilizer formulation and application ratios were maintained across the field trials to ensure consistency in the treatment comparisons. Field trials were conducted using the same treatments as defined for those employed in the pot experiments. The field experiment was conducted under typical regional climatic conditions for the tea-growing season, with average temperatures ranging from 24 °C to 32 °C, relative humidity between 75% and 90%, and regular monsoon rainfall. The inoculation was repeated every 30 days for all treatments except for the control and fertilizer treatments. The trial was conducted at the Rosekandy Tea Estate (24°67′00.83″ N and 92°69′87.32″ E), the Cachar district of Assam. Plants relied primarily on natural rainfall, supplemented with irrigation during dry spells. The plant growth parameters, plant enzyme activity, and soil enzyme activity were assessed after 90, 180, 270, and 360 days to evaluate the impact of the bacterial consortium, along with the NPK bio-stimulation on the plant growth and microbial diversity in the soil. Leaf samples were collected every 3 months for up to 12 months post-treatment to estimate the contents of chlorophyll (Chl-a, Chl-b) and carotenoids. Fresh leaves (0.5 g) were extracted in methanol and centrifuged, and the supernatant was analyzed spectrophotometrically at 663, 645, and 470 nm using standard equations [30]. The leaf relative water content (LRWC) was calculated using the formula LRWC (%) = [(FW − DW)/(TW − DW)] × 100, where FW, DW, and TW represent the fresh, dry, and turgid weights, respectively, after soaking in distilled water for 4–5 h. The experiment was conducted in triplicate [31].

### 2.6. Phenolic Content and Enzyme Activities in Tea Leaves

#### 2.6.1. Determination of Total Phenolic Content

The samples collected after 90, 180, 270, and 360 days were used to assess the effects of the four different treatments on the accumulation of phenolic compounds in two C. sinensis clones (S3A3 and TS491). The phenol content in aqueous tea extracts was measured using a modified Folin–Ciocalteu method. To 1 mL of tea extract, 2.5 mL of 10% Folin–Ciocalteu reagent and 2 mL of 7.5% Na_2_CO_3_ were added. The mixture was incubated for 15 min at room temperature, and the absorbance was measured at 765 nm. Gallic acid (1 mg/mL) served as the standard, with concentrations of 0.01, 0.02, 0.03, 0.04, and 0.05 mg/mL prepared in methanol. Measurements were taken in triplicate. The phenolic content was quantified using a gallic acid calibration curve and expressed as gallic acid equivalents (mg/g GAE) [32].

#### 2.6.2. Catalase Activity (CAT)

The sample (200 mg) was homogenized in 0.1 M phosphate buffer (pH 7.5) containing 1% pvp, 1 mM EDTA, and 10 Mm Beta-mercaptoethanol. The homogenates were centrifuged at 10,000 rpm for 20 min. Supernatant was collected for enzyme assays. The catalase was measured spectrometrically at 240 nm. The absorbance of the reaction solution was recorded every 30 s, and the change in absorbance of 0.01 units min^−1^ was defined as one unit of CAT activity [33].

#### 2.6.3. Glutathione Reductase (GR)

The reduced glutathione was determined by the method of Sarker et al. [34]. A homogenate containing glutathione was prepared by mixing 0.5 g of leaf sample with 2.5 mL of 5% trichloroacetic acid (TCA). The precipitated protein was centrifuged at 10,000 rpm for 10 min. The supernatant was used to estimate the glutathione (GSH). The 0.2 mL supernatant was made up to 2.5 mL with 0.2 M sodium phosphate buffer (pH 7.6), 0.5 mL of freshly prepared 5′,5-dinitrobenzoic acid (DTNB) solution was added, and the intensity of the yellow color that developed was measured in a spectrophotometer at 412 nm after 5 min of incubation. The value is expressed as nmol GSH g^−1^ sample. The experiment was conducted in triplicate.

#### 2.6.4. Superoxide Dismutase (SOD) Activity

To prepare the crude enzyme extract, 0.2 g of leaf sample was homogenized in 0.1 M phosphate buffer (pH 7.5) with 1% polyvinyl pyrrolidone (PVP), one mM EDTA, and 10 mM β-mercaptoethanol. The homogenate was centrifuged at 10,000 rpm for 20 min to obtain the crude enzyme. The superoxide dismutase (SOD) activity was assessed by adding 0.1 mL of the extract to 1.4 mL of 100 mM Tris-HCl buffer (pH 8.2), followed by 0.5 mL of 6 mM EDTA and 1 mL of 6 mM pyrogallol solution. The absorbance was measured at 420 nm after each 30 s interval for up to 3 min. One unit of enzyme activity was defined as the amount causing a 50% inhibition of pyrogallol auto-oxidation [33].

### 2.7. Soil Enzyme Activities

#### 2.7.1. Hydrolysis of Fluorescein Diacetate (FDA)

An amount of 1 g of soil was incubated with 50 mL of sodium phosphate buffer (pH 7.6) and 0.5 mL of FDA solution in a shaker at 24 °C for 1 h. The reaction was terminated by adding 2 mL of acetone. The mixture was centrifuged at 8000 rpm for 5 min, and the supernatant was filtered through Whatman No. 2 filter paper. The enzyme activity was quantified spectrophotometrically at 490 nm and expressed as fluorescein mg released g^−1^ dry soil [35].

#### 2.7.2. Dehydrogenase Activity (DA)

The dehydrogenase activity (DA) was evaluated by measuring the reduction of 2,3,5-triphenyltetrazolium chloride (TTC) to 2,3,5-triphenylformazan (TPF). One gram of soil was incubated with calcium carbonate (CaCO_3_), TTC, and distilled water at 30 °C for 24 h. The TPF was extracted with methanol, and the DA was quantified spectrophotometrically at 484 nm (formazan mg/g soil) [36].

#### 2.7.3. Soil Physicochemical Properties

The soil samples for all the treatments were collected at the depth of 0–20 cm in triplicate, and the physicochemical characteristics of the soil—comprising the pH, organic carbon (OC), total nitrogen (TN), total phosphorus (TP), and total potassium (TK)—were determined according to the standardized analytical protocols outlined by Lu [37]. The soil pH was measured by an alkaline hydrolysable technique [38] and a combination pH electrode (soil:water, 1:2.5 *w*/*v*). Kjeldahl digestion was used to quantify the total nitrogen (TN) and total organic carbon (TOC) [39], while the sodium carbonate technique and a NaOH melt flamer were used to measure the total potassium (TK) and total phosphorus (TP), respectively [40,41].

### 2.8. Bacterial Community Analysis of Rhizospheric Soil Before and After Bacterial Consortium During Field Trial Assay

Rhizospheric soil from the various treatments (bacterial consortium, 50% fertilizer + consortium, fertilizer, and control) was collected for bacterial community analysis. Soil samples (0.5 g) were extracted from the root zone, placed in cryo-vials, and stored in liquid nitrogen. DNA was isolated using the Nucleospin DNA kit (Düren, Germany), and amplicon libraries were prepared with the Nextera XT Index kit (Illumina, San Diego, CA, USA) targeting the V3–V4 region using 16S rDNA-specific primers (forward: 5′-GCCTACGGGNGGCWGCAG-3′; reverse: 5′-ACTACHVGGGTATCTAATCC-3′). Amplicons were sequenced on a MiSeq platform, Software version 2.6 (Illumina, San Diego, CA, USA) with a 2 × 300 bp paired-end method. Quantitative Insights into Microbial Ecology (QIIME 1) was used to examine the paired-end sequences [42]. Sequences were analyzed with QIIME 1 and clustered into operational taxonomic units (OTUs) at 97% similarity using Uclust (USEARCH) version: 5.2.236, and the taxonomy was assigned using the GreenGenes Database. By matching the readings against the Green Genes Database (version 13.8), taxonomy was assigned to the OTUs. Functional prediction was performed using PICRUSt1, with the KEGG pathways categorized at level 3. Comparisons were made between the treated and control samples to assess the microbial community structure and functional profiles [43]. In the figures related to the bacterial community analysis, the treatment acronyms represent the different experimental conditions analyzed before and after treatment for the two tea clones. For the S3A3 tea clone, ISS1 (control), ISS2 (consortium), ISS3 (50% fertilizer + consortium), and ISS4 (fertilizer) indicate the samples collected before treatment, while FSS1 (control), FSS2 (consortium), FSS3 (50% fertilizer + consortium), and FSS4 (fertilizer) represent the samples collected after treatment. Similarly, for the TS491 tea clone, ITS1 (control), ITS2 (consortium), ITS3 (50% fertilizer + consortium), and ITS4 (fertilizer) denote the samples collected before treatment, and FTS1 (control), FTS2 (consortium), FTS3 (50% fertilizer + consortium), and FTS4 (fertilizer) correspond to the samples collected after treatment.

### 2.9. Statistical Analysis

Data on the effects of the different treatments on the tea plants are presented as means ± standard deviations from three independent assays with three replicates each. A one-way ANOVA was performed with the treatment as the independent variable and the measured parameters (e.g., plant growth or microbial indices) as the dependent variables, using SPSS (version 20) with a significance threshold of *p* ≤ 0.05. The α-diversity was assessed in PAST (v4.03) using the Richness (S), Shannon (H), and Chao-1 indices. The β-diversity was analyzed using the Whittaker index in PAST (v4.03). Functional gene analysis was conducted using STAMP (v2.1.3). Figures were generated based on the ANOVA followed by Tukey–Kramer post hoc tests at *p* ≤ 0.05. To assess the interrelationships among the dominant bacterial genera, pairwise Spearman’s rank correlation coefficients were calculated based on their relative abundance profiles across samples. Only statistically significant correlations (*p* < 0.05), corrected for multiple comparisons using the Benjamini–Hochberg false discovery rate (FDR) method, were retained for the network construction. Correlation networks were generated with positive and negative correlations represented by red and blue edges, respectively. The width of each edge was proportional to the strength of the correlation coefficient. Network visualization was performed using Cytoscape (version 3.9), with the nodes representing bacterial genera and the edges indicating the significant correlations between them [44]. Separate networks were constructed for the tea clones S3A3 and TS49.

## 3. Results and Discussion

### 3.1. Microorganisms and Their PGP Attributes

The increased use of chemical fertilizers and pesticides in tea cultivation has had an adverse impact on plant health, the environment, and public health [45,46]. Consequently, there is a shift toward organic methods that utilize local microbial isolates. The use of bacterial consortia as a sustainable alternative to NPK (nitrogen, phosphorus, potassium) chemical fertilizers in tea gardens is gaining attention for its potential to enhance plant growth-promoting (PGP) attributes and improve the soil microbial diversity. This approach aligns with the increasing need for sustainable agricultural practices that minimize environmental impacts while maintaining crop yields and quality. In this study, 81 rhizobacterial isolates obtained from the rhizospheric soil of tea plants were screened for their key plant growth-promoting (PGP) traits, including their phosphate solubilization, siderophore production, indole-3-acetic acid (IAA) production, ACC deaminase activity, ammonia production, and hydrogen cyanide (HCN) production. Among these isolates, 46 were found to solubilize phosphate, with the highest solubilization observed in *Bacillus thuringiensis* WJB5 (57.77 µg/mL) and *S. marcescens* CIC2 (57.09 µg/mL). Siderophore production was the highest in *S. marcescens* WJB6 with 66.91%, while 182.12 µg/mL indole-3-acetic acid (IAA) was recorded in *S. marcescens* CIC2. *L. fusiformis* BS14 and *S. marcescens* PH3 exhibited the highest ACC deaminase activities: 100.05 nmol α-ketobutyrate mg^−1^ protein h^−1^ each.

Previous studies have reported the prevalence of *Bacillus* spp. in tea rhizospheres with their ability to produce indole-3-acetic acid (IAA), a key growth-promoting substance [2]. IAA production by tea rhizosphere isolates, such as *L. fusiformis* AB332 and *B. thuringiensis* AB341, with respective yields of 48.83 µg mL^−1^ and 28.50 µg mL^−1^, has been suggested to be important for growth improvements [47,48]. The present study identified *S. marcescens* (CIC2 and PH3), *L. fusiformis* BS14, and *B. thuringiensis* WJB5 as superior IAA producers, with markedly higher IAA yields of 182.12 µg mL^−1^, 39.10 µg mL^−1^, 90.62 µg mL^−1^, and 36.55 µg mL^−1^, respectively. Additionally, other isolates, including *Bacillus* spp. (SS2) and *Serratia* spp. (FS1 and WJB6), also demonstrated enhanced IAA production and multiple plant growth-promoting attributes. *L. fusiformis* BS14 and *S. marcescens* PH3 exhibited the highest ACC deaminase activities (100.05 nmol α-ketobutyrate mg^−1^ protein h^−1^ each). Additionally, 53 isolates produced ammonia, and 25 were capable of producing hydrogen cyanide (HCN). Out of 60 rhizobacterial isolates exhibiting at least one plant growth-promoting (PGP) trait, 26 demonstrated a full spectrum of key plant growth-promoting (PGP) attributes. From this subset, eight isolates were selected as the most promising candidates for further application. These strains were chosen based on their superior PGP efficiency and mutual compatibility. Importantly, they exhibited complementary functional traits, making them ideal for the formulation of an effective and synergistic multispecies consortium aimed at enhancing the growth of tea plants. The detailed PGP profiles of these eight selected isolates are summarized in Table 1.

The positive effects on shoot and leaf counts, chlorophyll contents, and enzymes are known to enhance plant growth by increasing the nutrient availability through root exudate buildup, phosphate solubilization, and nitrogen accumulation activities, and the nutrient uptake in tea plants, which could be beneficial for host plants [49,50].

### 3.2. Characterization of Rhizobacterial Isolates

Selected bacterial isolates were characterized based on 16S rRNA sequence homology. The sequences were analyzed using the BLAST tool, leading to the identification of the following isolates: *L. fusiformis* BS14 (NCBI accession no. OQ799287.1), *Bacillus* sp. SS2 (OQ569706), *Bacillus thuringiensis* WJB5 (OQ799921.1), and five strains of *S. marcescens* (CIC2: OQ799288.1; FS1: OQ789244.1; PH3: OQ799289.1; SRH1: OQ799290.1; and WJB6: Q799291.1). A neighbor-joining phylogenetic tree constructed using MEGA 11, employing the Tamura–Nei model with the bootstrap analysis of 1000 replicates, is shown in the Supplementary Materials (Figure S1). The scale bar represents differences in the base compositions among the sequences.

### 3.3. Pot and Field Trial Assays with C. sinensis Varieties S3A3 and TS491

#### 3.3.1. Effect of Rhizobacteria on the Growth of Tea Plants in Greenhouse and Field Conditions

The impact of a rhizobacterial consortium on tea plants was assessed through a six-month pot trial and a one-year field trial at the Rosekandy Tea Estate, Cachar, Assam. The consortium’s effects on the plant growth parameters, chlorophyll content, enzyme activities, and microbial diversity were evaluated. The results of our study showed significant improvements in the shoot lengths, root lengths, leaf numbers, and water contents in the tea varieties (S3A3 and TS491) compared to the fertilizer-treated and control tea plants. Enhanced enzyme activities related to nitrate assimilation and nutrient use efficiency were observed, suggesting that PGPB treatment supports plant growth and yields [51]. Additionally, microbial phosphorus solubilization has gained importance due to the rising costs of synthetic phosphates, with bacteria converting immobilized phosphorus into soluble forms [52]. PGPB also contribute to plant development by synthesizing phytohormones, solubilizing inorganic phosphates, and increasing the iron availability through the production of siderophores and volatile compounds [16,53].

The pot trial with the different treatments showed that the microbial consortia significantly enhanced the growth performance of the *C. sinensis* clones S3A3 and TS491, as shown in Supplementary Figure S2A,B. The plant growth parameters, including the root and shoot lengths, leaf count, water content, and chlorophyll concentration, were assessed systematically to ensure a precise evaluation of the bacterial efficiency over the fertilizer dosage and control treatments (refer to Supplementary Table S1). The results of the plant and soil enzyme activities are provided in Supplementary Figures S3 and S4.

**Figure 1:**
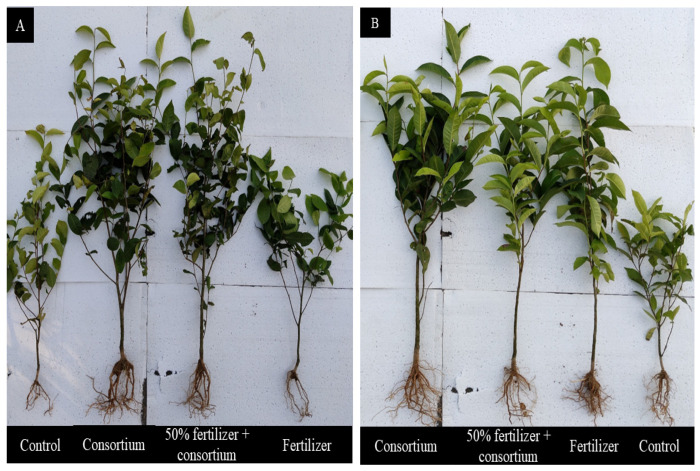
Effects of different treatments on growth of *C. sinensis* varieties (**A**) S3A3 and (**B**) TS491 during field trials.

In the field trial, all the treatments exhibited comparable initial shoot lengths (14–15 cm) for both *C*. *sinensis* clones, S3A3 and TS491 (Figure 1A,B). After 12 months, plants treated with the bacterial consortium showed the highest shoot lengths (86.27 cm in S3A3 and 88.5 cm in TS491), whereas the 50% fertilizer + consortium treatment resulted in intermediate growth. The fertilizer (only) treatment and the control exhibited the lowest shoot elongation. Similarly, the initial root lengths were consistent across the treatments (4–5.5 cm), but after 12 months, the consortium treatment led to the most substantial root development (14.1 cm in S3A3 and 13 cm in TS491), while the other treatments exhibited moderate root growth. At the start of the experiment, the numbers of leaves per plant were similar across all the treatments (6–7). However, by the end of the trial, the plants treated with the bacterial consortium displayed significant increases in their leaf numbers (96.33% in S3A3 and 99.67% in TS491). The 50% fertilizer + consortium treatment also enhanced the leaf production, although to a lesser extent than the consortium alone. In contrast, the control and fertilizer (only) treatments exhibited the lowest leaf counts. The water content analysis revealed that the consortium-treated plants retained the highest water contents (78.14% in S3A3 and 76.24% in TS491). The 50% fertilizer + consortium treatment also maintained relatively high water retention (69%), whereas the control and fertilizer-only treatments exhibited the lowest values (54–61%).

Previous findings have demonstrated that bacterial inoculation increases the nutrient bioavailability, remediating the soil structure by improving its aggregation and stability [54]. In our study, we also found that the bacterial consortium improved the soil physicochemical properties. Bacterial strains produced bioactive compounds, mobilizing the fixed or unavailable P, K, and Fe in the soil, which positively influences soil fertility [55]. The change in the soil physicochemical properties supports our findings. Different studies have shown that rhizobacteria from soil rhizospheres significantly improve the plant growth and yields of various tea cultivars [3,13]. Kaymak et al. [56] observed that amendment with a bacterial consortium significantly enhanced plant growth, increasing the root length by 41.3% and the shoot length by 17.5% compared to the untreated control after 60 days of sowing, at *p* < 0.05. Similar to this, the results from our experiments revealed that the tea plants inoculated with the consortium exhibited enhanced growth, as reflected in the increased shoot lengths of 86% and 88% and root lengths of 14% and 13% in S3A3 and TS491, respectively, when compared to the control groups and 50% fertilizer + consortium-amended treatment. Rhizobacterial applications also protect crops from fungal diseases and boost agricultural productivity [46,57]. Our study found a strong correlation between the PGPB-induced growth promotion and enzyme activity in the plant leaves of the consortium-treated plants as compared to the fertilizer-treated and control plants. Different plant growth parameters increased significantly in the microbial-treated plants with a 50% reduction in the NPK amendment. The bacterial consortium-amended plants showed a maximum leaf count compared to the NPK treatment and control (Table 2).

#### 3.3.2. Rhizobacterial-Mediated Regulation of Plant Enzymatic Activity During Field Trial Study

**Figure 2:**
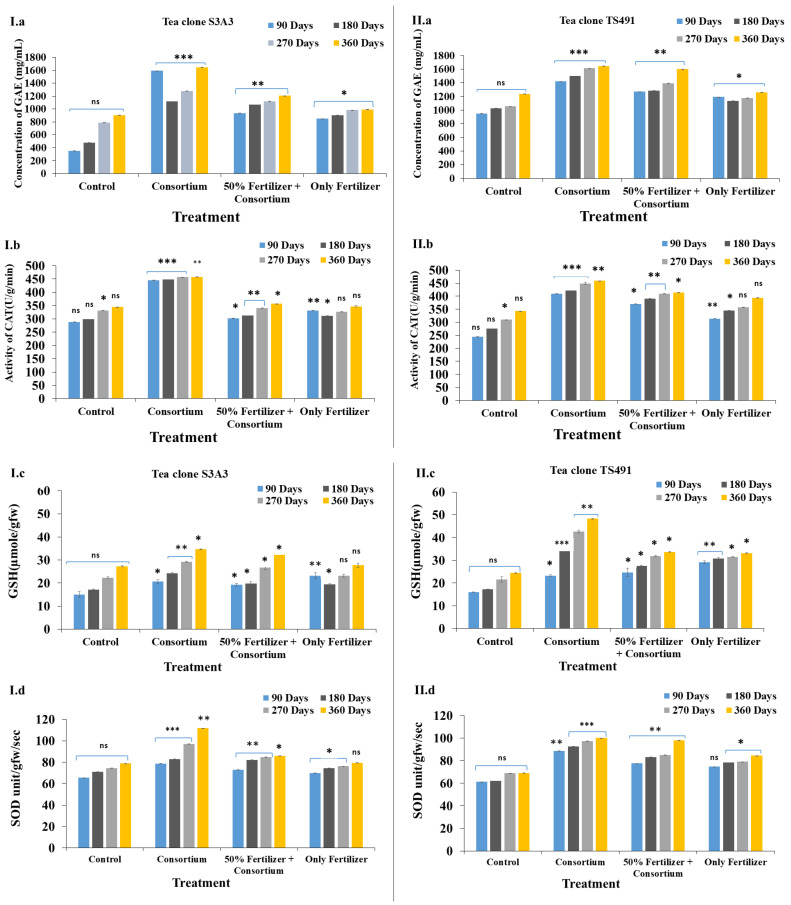
Evaluation of the enzyme activities of the tea plants during the field trial experiment to show the effects of the different treatments in the two tea clones: S3A3 and TS491: Phenolic content (**I.a**,**II.a**), CAT activity (**I.b**,**II.b**), glutathione reductase (**I.c**,**II.c**), and SOD activity (**I.d**,**II.d**). Values bearing different signs (*, **, ***) differ significantly. * *p* ≤ 0.05 (significant), ** *p* ≤ 0.01 (highly significant), *** *p* ≤ 0.001 (very highly significant).

Field trials conducted over 90 to 360 days demonstrated a progressive effect of the PGPB treatment on the tea plants, as measured by the phenol content, superoxide dismutase (SOD) activity, glutathione (GSH) content, and catalase (CAT) activity. Gallic acid equivalents (GAEs), a key indicator of the total phenolic content, play a vital role in plant defense and antioxidant activity. PGPB inoculation has been shown to enhance polyphenol levels, SOD activity, and the overall antioxidant capacity, thereby improving plant resilience to microbial stress [58,59]. In the present study, the highest phenolic content was observed in the consortium-treated plants, at 1643.6 mg GAE/mL in S3A3 and 1646.93 mg GAE/mL in TS491. In contrast, the control and fertilizer-only treatments showed lower GAE levels: 905.6 mg/mL and 992.93 mg/mL (control) and 1238.26 mg/mL and 1260.93 mg/mL (consortium), respectively (Figure 2(I.a,II.a)). These findings align with those of Mittler [60], who also reported increased phenolic accumulation under consortium application compared to individual strains.

Catalase is another crucial antioxidant enzyme that decomposes hydrogen peroxide into water and oxygen, thereby protecting plant cells from oxidative damage, and is crucial for oxidative stress management [61,62]. The PGPB consortium treatment increased the polyphenolic concentrations, enhancing the ROS scavenging and stress tolerance in the tea plants [63]. Inoculation with PGPB significantly enhanced the growth of the tea plants, leaf production, and antioxidant enzyme activities. The catalase (CAT) activity was the highest in the consortium-amended treatments, reaching approximately 458 U/g/min in both S3A3 and TS491, compared to the 50% fertilizer + consortium treatment, which had lower CAT activities, ranging from 357 to 415 U/g/min in S3A3 and TS491, respectively. In contrast, the control (344.63 U/g/min in S3A3 and 342.63 U/g/min in TS491) and fertilizer (only) (347.16 U/g/min in S3A3 and 394 U/g/min in TS491) treatments exhibited significantly lower CAT activities. The increased catalase activities in the consortium-treated plants suggests a strengthened antioxidative defense mechanism, potentially enhancing the stress tolerance in *C. sinensis* (Figure 2(I.b,II.b)). These findings are consistent with previous studies, which have shown that bacterial consortia positively influence catalase production, with reported values of 145 U/mg protein [64].

The glutathione (GSH) concentration in the two tea clones (S3A3 and TS491) over different time periods (90, 180, 270, and 360 days) notably exhibited the highest glutathione contents in the consortium-treated plants, with values of 34.67 µmole/gfw and 42.67 µmole/gfw, respectively. The control treatment with (27.22 µmole/gfw in S3A3 and 24.44 µmole/gfw in TS491) and only-fertilizer treatments (27.77 µmole/gfw in S3A3 and 33.16 µmole/gfw in TS491) showed lower GSH concentrations, indicating reduced antioxidant capacities. The control plants exhibited the lowest glutathione levels, which are given in Figure 2(I.c,II.c). The glutathione S-transferase (GST) contents also increased in the consortium-treated plants, with peak values of 34.67 µmole/gfw and 42.67 µmole/gfw in S3A3 and TS491, respectively.

Superoxide dismutase (SOD) is another critical enzyme that protects cells from oxidative stress by dismutating superoxide radicals into less harmful molecules. The consortium treatment resulted in the highest SOD activities (S3A3: 111.7 unit/gfw/s; TS491: 100.06 unit/gfw/s), followed closely by the 50% fertilizer + consortium (S3A3: 86.05 unit/gfw/s; TS491: 98.11 unit/gfw/s) in both tea clones. The control and fertilizer (only) treatments showed moderate SOD activities, which were lower compared to the consortium treatments (S3A3: 78.91 unit/gfw/s; TS491: 69.08 unit/gfw/s; and S3A3: 79.50 unit/gfw/s; TS491: 84.47 unit/gfw/s) (Figure 2(I.d,II.d). Our findings demonstrate that the application of a PGPB consortium significantly enhances plant growth and antioxidant enzyme activities while simultaneously reducing the dependence on chemical fertilizers [59,65,66]. In the field study, S3A3 had higher SOD activity, indicating a stronger antioxidative defense, whereas TS491 showed higher GST activity, suggesting better detoxification. The CAT activities and phenolic contents were similar in both clones, reflecting comparable oxidative stress tolerances.

The integration of PGPB with nitrogen (N) and phosphorus (P) fertilizers has been shown to promote more sustainable yield outcomes compared to the exclusive reliance on chemical fertilizers [67]. In our study, the observed increase in enzyme activities in the PGPB-treated plants was particularly important for strengthening metabolic and defensive pathways. Enhanced antioxidant enzyme activity plays a crucial role in mitigating oxidative stress, improving plant resilience against abiotic stressors such as drought and soil contamination [68]. This is highly relevant in the context of climate change (SDG 13: Climate Action), where environmental stressors are becoming more prevalent, impacting agricultural productivity. Furthermore, the PGPB application supports SDG 2 (Zero Hunger) by promoting sustainable agricultural practices that enhance crop productivity while reducing the dependency on synthetic fertilizers. Excessive chemical fertilizer use contributes to soil degradation, water pollution, and greenhouse gas emissions, making PGPB-based biofertilization a viable alternative for achieving SDG 12 (Responsible Consumption and Production) and SDG 15 (Life on Land). The development of stable PGPB formulations is a promising strategy for sustainable tea cultivation, ensuring long-term soil health, biodiversity conservation, and climate-resilient agriculture. Developing stable PGPB formulations is a promising strategy for sustainable tea cultivation [69].

#### 3.3.3. Effect of Bacterial Consortium on Soil Enzyme Activities During Field Trials

Enzyme activities and soil nutrients are closely linked, with soil enzymes produced by microbial communities regulating macro- and micronutrients and serving as early indicators of the soil health [70]. In field conditions, the highest fluorescein diacetate activity was noted in the plants treated with the bacterial consortium (S3A3: 9.93 mg fluorescein/g/g soil; TS491: 11.11 mg fluorescein/g/g soil). This was followed by the treatment combining 50% fertilizer with the consortium (S3A3: 8.05 mg fluorescein/g soil; TS491: 8.74 mg fluorescein/g soil) and the fertilizer (only) treatment (S3A3: 6.39 mg fluorescein/g soil; TS491: 7.76 mg fluorescein/g soil) across all time points, indicating higher microbial activity and soil enzymatic function (Supplementary Figure S5(I.a,II.a). The fertilizer (only) and control treatments had lower fluorescein production, suggesting reduced microbial activity and lower organic matter decomposition. The control and fertilizer (only) treatments had comparatively lower fluorescein levels, reinforcing the importance of microbial inoculation in enhancing the soil biochemical activity.

High enzyme activity, including dehydrogenase, is typical in organic farms and correlates with increased microbial biomass and diversity [71]. Our findings support this, showing elevated dehydrogenase activity in the soil treated with the microbial consortia. In contrast, excessive chemical fertilizers can reduce enzyme activity, consistent with the lower enzyme levels observed on traditional farms [72]. Since the dehydrogenase activity is meant to reflect the entire spectrum of oxidative activity of the soil microbiota, it is a widely recognized measure of microbial activity [73]. Fertilizer types affect the soil conditions and microbial dynamics, influencing enzyme activity [74]. Tea plants may also influence rhizosphere microbial communities, shaping bacterial assemblages [75]. The dehydrogenase activities of the various treatments in the field trial were the highest for the consortium treated-plants for both tea clones (S3A3 and TS491), with the values 0.29 formazan mg/g soil and 0.25 formazan mg/g soil, respectively, across all the time points, indicating a high microbial respiration rate and enhanced soil health. The 50% fertilizer + consortium treatment also showed increased formazan production, suggesting that the microbial activity was still improved despite the partial reduction in the dosage of fertilizer. The control and fertilizer (only) treatments had the lowest formazan values, indicating lower microbial activities, as shown in Supplementary Figure S5(I.b,II.b). The increase in the hydrolysis of fluorescein diacetate (FDA) and dehydrogenase activity observed in the present study in the consortium-amended rhizospheric soil, compared to the fertilizer-treated and control soils, confirms that the bacterial consortium can overcome the use of harmful fertilizers through bacterial formulation.

We selected two different tea clones for this study based on their widespread cultivation in the experimental region and their agronomic relevance to local tea plantations. Both varieties are commonly used in regional tea production and are therefore considered suitable models for evaluating the efficacy of a plant growth-promoting bacterial consortium under field conditions. Although both showed responses relative to the control, the extent of the effects varied between them. The pot and field trials with the PGPB consortium revealed clone-specific responses: TS491 showed greater shoot elongation and higher carotene content, indicating stronger photoprotection, while S3A3 exhibited enhanced root growth and higher chlorophyll levels, reflecting superior photosynthetic potential. Microbial profiling revealed that TS491 favored shoot growth linked to *Firmicutes* and *Bacillus*, while S3A3 supported root development associated with *Acidobacteria* and *Gemmataceae*. These findings suggest genotype-specific responses to microbial inoculation, with clonal traits influencing plant–microbe interactions. Earlier, it was also suggested that different tea clones respond variably to fertilizers and bioinoculants due to genetic and physiological differences. Studies show that the growth, yield, and nutrient uptake vary significantly among cultivars under different fertilizer regimes. For instance, a field study with ten tea cultivars under varying nitrogen levels revealed distinct growth and biomass responses, with some clones showing greater tolerance to low nitrogen [76]. This highlights significant cultivar–nitrogen interactions and non-uniform fertilization responses across tea clones. In another study, it was observed that the tea plant’s response to fertilizer was more influenced by the clone type than the plant age. The genotype had a greater impact on productivity, with the yield response to nitrogen largely determined by whether the plant was a seedling or clonal cultivar [77]. Previous studies on the tea clones TV1, TV19, and TV20 demonstrated that PGPB had varying effects across clones, with TV19 showing greater increases in the root and shoot lengths and biomass compared to TV1 and TV20 [78]. Similarly, our study on the tea clones S3A3 and TS491 revealed significant differences in the nutrient uptake and root-associated traits, reinforcing these earlier findings.

#### 3.3.4. Changes in Soil Physiochemical Properties After the Treatment

Soil chemistry is a determining factor in soil fertility [79]. The data on the soil physicochemical properties—including the pH, organic carbon (OC), and major nutrients (nitrogen, phosphorus, and potassium)—revealed significant improvements following the application of a bacterial consortium to the rhizosphere soil. The soil pH was slightly increased for both tea clones—S3A3 and TS491—with the consortium treatment, whereas the treatments combining 50% fertilizer with the consortium showed no notable change. Further, the consortium treatment caused increases in the organic carbon content of 22.70% in the S3A3 soil and of 11.35% in the TS491 soil, as compared to the control. Also, the total nitrogen increased by 25% in the S3A3 soil samples and by 24.27% in the TS491 soil samples. Similarly, the total phosphorus rose by 12.21% in the S3A3 soil samples and by 10.85% in the TS491 soil samples. The total potassium content also showed substantial increases: 44.56% in the S3A3 soil samples and 44.12% in the TS491 soil samples. Detailed results are provided in Supplementary Table S2. Previous work has reported on the role of PGPR in improving the physicochemical properties of soils through several mechanisms, including the fixation of nitrogen, the solubilization of phosphorus and potassium, and the release of exopolysaccharides and siderophores, which are important for soil stabilization [80,81]. Organic carbon is the primary nutrient responsible for the physical, chemical, and biological characteristics of soil [82].

#### 3.3.5. Effect of Rhizobacteria on Bacterial Community Analysis During Field Trial Assay

**Figure 3:**
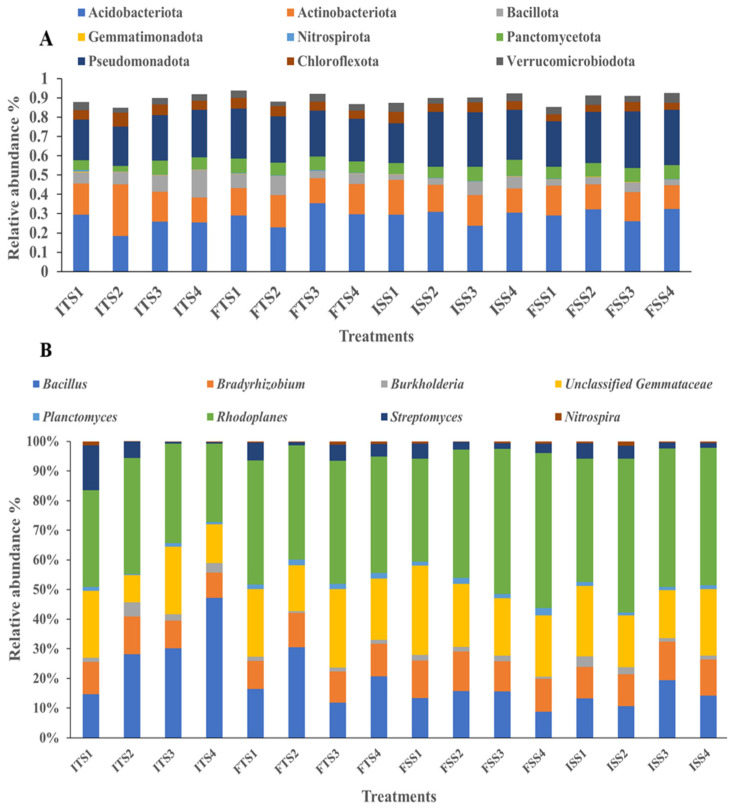
Bacterial communities at the (**A**) phylum level and (**B**) genus level for the different treatments (samples collected before and after treatments): ISS1 (control), ISS2 (consortium), ISS3 (50% fertilizer + consortium), ISS4 (fertilizer) represent data for “before treatment”, while FSS1 (control), FSS2 (consortium), FSS3 (50% fertilizer + consortium), and FSS4 (fertilizer) represent data for “after treatment” as analyzed for the S3A3 tea clone. Similarly, ITS1 (control), ITS2 (consortium), ITS35 (0% fertilizer + consortium), and ITS4 (fertilizer) represent data for “before treatment” and FTS1 (control), FTS2 (consortium), FTS3 (50% fertilizer + consortium), and FTS4 (fertilizer) represent data for “after treatment” as analyzed for the TS491 tea clone.

Different researchers have shown that soil microbial populations vary with fertilization treatments [68,69]. In this study, the soil treated with the consortium showed a significant increase in beneficial bacteria. In both tea clones, S3A3 and TS491, the microbial communities were dominated by bacteria, followed by Archaea. In the control soil with no bacterial inoculation, the predominant phyla were *Acidobacteria* (28%), *Proteobacteria* (23%), *Actinobacteria* (16%), *Planctomycetes* (6%), and *Bacteroidetes* (4%). With the consortium treatment, the proportion of *Acidobacteria* increased to 32%, while *Proteobacteria* and *Actinobacteria* remained the most prominent. The fertilizer amendment with the bacterial consortium shifted the dominance to *Proteobacteria* (29%), followed by *Acidobacteria* (26%) and *Actinobacteria* (15%). In the treated TS491 soil, the phyla distribution was *Acidobacteria* (23%), *Proteobacteria* (24%), *Actinobacteria* (17%), *Firmicutes* (10%), and *Planctomycetes* (6%). Overall, the application of the bacterial consortium enhanced the relative abundances of *Acidobacteria*, *Proteobacteria*, and *Actinobacteria* across both tea varieties (Figure 3A). Previous research reported that the most dominant bacterial phyla in tea plantations are *Acidobacteria*, *Proteobacteria*, *Chloroflexi*, *Actinobacteria*, and *Firmicutes* [83].

At the genus level, the application of the bacterial consortium increased the abundances of beneficial genera such as *Rhodoplanes* and unclassified *Gemmataceae*, *Bacillus*, *Bradyrhizobium*, *Nitrospira*, and *Planctomyces* compared to the uninoculated control. However, the Streptomyces abundance decreased with the consortium treatment. The abundance of *Bacillus* also decreased in the soils treated with fertilizer for both tea varieties, S3A3 and TS491, but increased in the soils treated with the consortium. These results suggest that the consortium application enriched beneficial bacteria, thereby enhancing their dominance and reducing the dominance of less impactful genera (Figure 3B). The consortium treatments shifted the microbial community, with *Acidobacteriota*, *Gemmatimonadota*, *Pseudomonota*, *Actinobacteriota*, *Nitrospirota*, *Chloroflexota*, *Bacillota*, *Planctomycetota*, and *Verrucomicrobiota* as the dominant phyla. Earlier, *Proteobacteria*, *Actinobacteria*, *Bacteroidetes*, *Acidobacteria*, and *Chloroflexi* were observed as the dominant phyla in both tea rhizospheres [84]. The consortium treatments influenced the bacterial community structure, affecting the plant growth and soil health. The rhizosphere’s microbial composition and diversity are directly linked to the functional gene abundance and soil health [85]. A previous study shows that *Bacillus* species, particularly *B*. *subtilis* and *B. mycoides*, are prominent in the tea plant rhizobiome and remain effective under adverse conditions [86]. These *Bacillus* strains exhibit antagonistic activity against fungal pathogens and disrupt fungal mycelium formation. *Bradyrhizobium* enhances plant growth [87], while *Streptomyces* species promote plant growth and control plant diseases through antibiotic production and various biological activities [88,89]. *Gemmatimonas* plays a crucial role in the carbon cycle by decomposing organic matter [90,91], and *Burkholderia*, a β-proteobacterium, supports plant growth in diverse ecological niches [92]. The control treatment yielded a bacterial network with short path lengths and low modularity, suggesting rapid yet unstable propagation across the network. This instability could disrupt key microbial communities involved in the soil carbon, nitrogen, and phosphorus cycles, potentially affecting *C. sinensis* growth. In contrast, the microbial consortium enhanced the network stability by increasing the number and size of the network modules, resulting in more significant hubs and inter-module linkages. This suggests that a higher number of ecological niches and stronger bacterial connections can improve the community stability and the adaptability to environmental changes.

**Figure 4:**
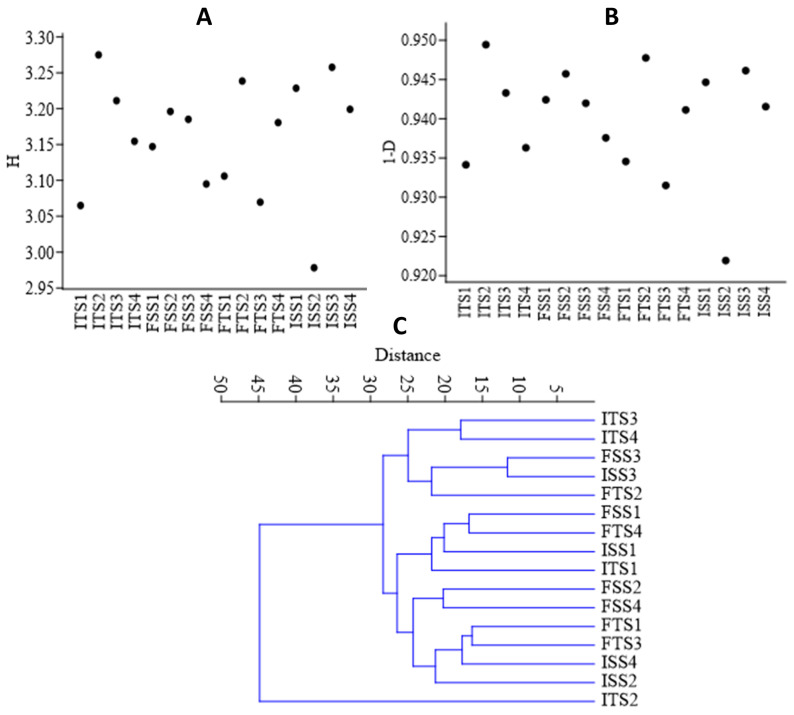
(**A**) Shannon (H) index, (**B**) Simpson diversity index, and (**C**) beta diversity analysis of the samples using a Whittaker index distance matrix. The treatments include the control, consortium, 50% fertilizer + consortium, and fertilizer. For the S3A3 tea clone, these are denoted as ISS1, ISS2, ISS3, and ISS4 for the samples collected before treatment, and as FSS1, FSS2, FSS3, and FSS4 for the samples collected after treatment. Similarly, for the TS491 tea clone, ITS1–ITS4 represent the respective treatments before the application, while FTS1–FTS4 correspond to the samples after treatment. Detailed treatment descriptions are provided in the main text.

The bacterial diversity was assessed using alpha diversity metrics via rarefaction to estimate the species richness from amplicon sequence variants (ASVs). The consortium-inoculated soils (ISS2, FSS2, ITS2, FTS2) showed higher abundances of the selected bacteria, whereas the uninoculated soils (ISS1, FSSI, ITS1, FTS1) exhibited greater overall diversity. The Shannon and Simpson indices were used to quantify the diversity, with the Shannon index reflecting the species diversity (Figure 4A) and the Simpson index estimating the species richness (Figure 4B). The beta diversity, based on Whittaker distances, indicated that the uninoculated control was distinct, while the other samples shared common genera (Figure 4C).

**Figure 5:**
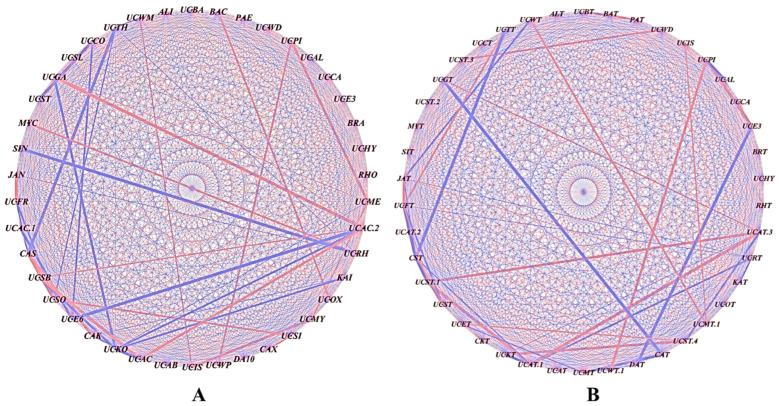
Co-occurrence analysis illustrating the strength of the correlations between the different bacterial genera: (**A**) S3A3 and (**B**) TS491. The thickness of the lines between the nodes denotes the strength (*p* < 0.05). Red lines indicate a positive correlation among the abundances of the linked taxa, whereas the blue lines indicate a negative correlation. The network was visualized using Cytoscape (Version 3.9).

The influence of the bacterial genera on the community structure was analyzed by correlating their relative abundances in the rhizobial-treated plants versus the untreated controls. Significant correlations (*p* ≤ 0.05) were assessed using Pearson’s coefficient, with the nodes and edges depicting the bacterial connections. The red lines denote strong positive correlations, while the blue lines indicate negative correlations. Positive correlations were observed between genera such as unclassified *Thermogemmatisporaceae* (UCTH) and *Mycobacterium* (MYC), and between *Paenibacillus* (PAE) and unclassified *Actinomycetales* (UCAC2). Negative correlations were seen between *Alicyclobacillus* (ALI) and unclassified *Caulobacteraceae* (UCCA), and between *Sinomonas* (SIN) and unclassified *Pirellulaceae* (UCPI) (Figure 5A). The rhizobial treatment increased the abundances of mutually dependent bacterial groups, which were less prevalent in the uninoculated control.

Positive correlations were found between unclassified WMSP1 (UCWT) and *Methylocystaceae* (UCMT.1); *Isosphaeraceae* (UCIS) and *Methylocystaceae* (UCMT.1); *Streptomycetaceae* (UCST3) and WD2101 (UCWD); *Pirellulaceae* (UCPI) and WPS-2 (UCWT.1); *Thermogemmatisporaceae* (UCTT) and *Frankiaceae* (UCFT); and *Gaiellaceae* (UCGT) and *Actinomycetales* (UCAT.3) (Figure 5B). Negative correlations were observed between Janibacter (JAT) and WMSP1 (UCWT); *Candidatus solibacter* (CST) and *Thermogemmatisporaceae* (UCTT); *Candidatus xiphinematobacter* (CAT) and *Gaiellaceae* (UCGT); DA101 (DAT) and *Ellin*329 (UCE3); and *Actinomycetales* (UCAT.3) and *Isosphaeraceae* (UCST.4). The rhizobial treatment enhanced the abundances of interdependent bacterial groups that were less prevalent in the uninoculated control.

#### 3.3.6. Effect of Rhizobacteria on Bacterial Predicted Functions During Field Trial Assay

**Figure 6:**
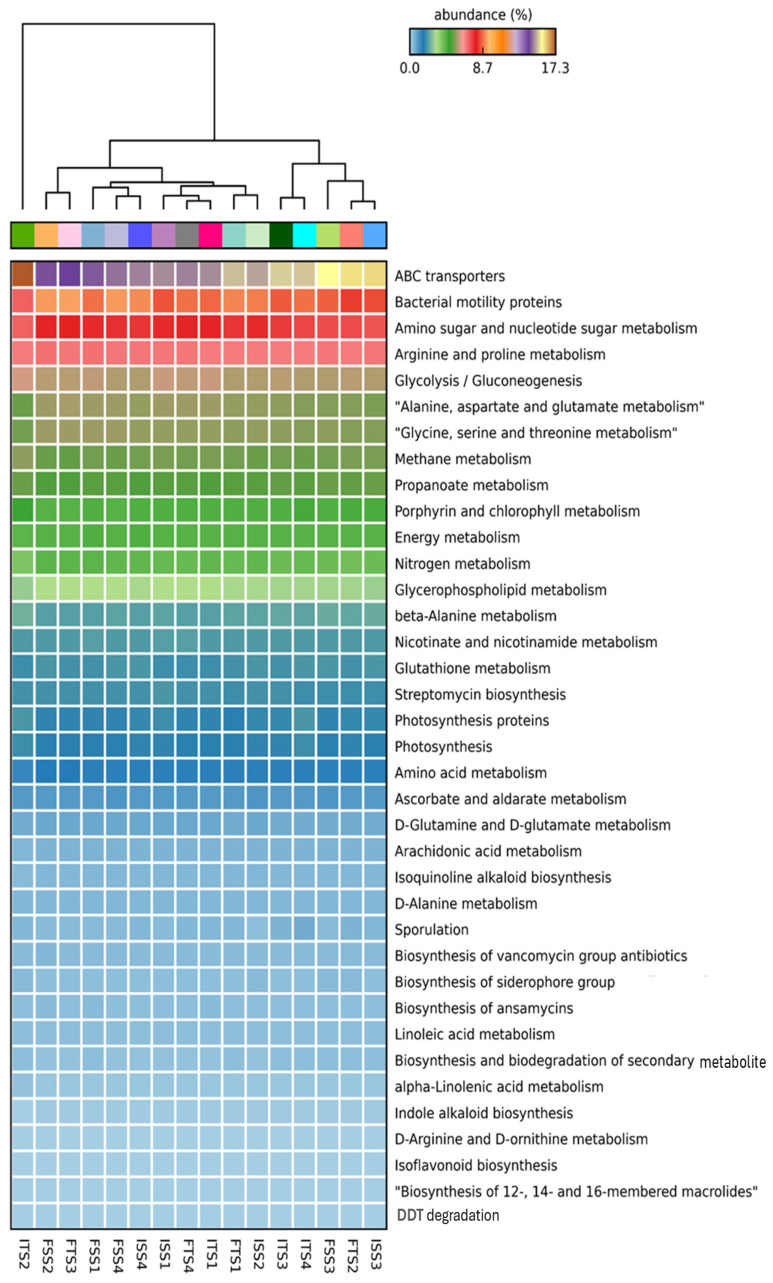
KEGG level 3 functional groups of rhizospheric soil communities of S3A3 and TS491 that were varied after the application of the bacterial consortium. Only those with relative abundances greater than 0.1% are shown. The treatments include the control, consortium, 50% fertilizer + consortium, and fertilizer. For the S3A3 tea clone, these are denoted as ISS1, ISS2, ISS3, and ISS4 for the samples collected before treatment, and as FSS1, FSS2, FSS3, and FSS4 for the samples collected after treatment. Similarly, for the TS491 tea clone, ITS1–ITS4 represent the respective treatments before application, while FTS1–FTS4 correspond to the samples after treatment. Detailed treatment descriptions are provided in the main text.

**Figure 7:**
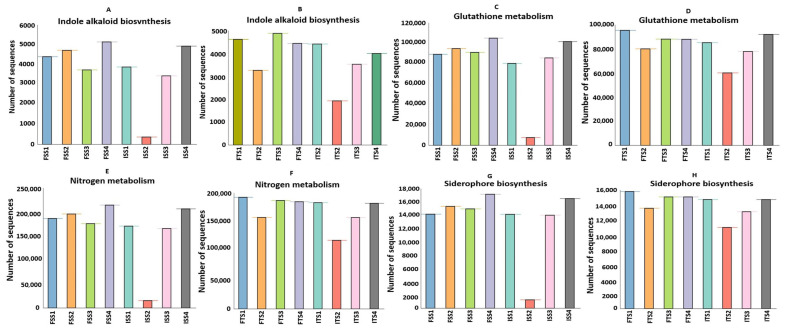
Predicted increases in the abundances of functional genes in the consortium-treated soils of S3A3 and TS491, as compared to the control for the genes responsible for (**A**,**B**) indole alkaloid biosynthesis, (**C**,**D**) glutathione metabolism, (**E**,**F**) nitrogen metabolism, and (**G**,**H**) the biosynthesis of siderophore group non-ribosomal peptides. The figures are significant at *p* ≤ 0.95. The treatments include the control, consortium, 50% fertilizer + consortium, and fertilizer. For the S3A3 tea clone, these are denoted as ISS1, ISS2, ISS3, and ISS4 for the samples collected before treatment, and as FSS1, FSS2, FSS3, and FSS4 for the samples collected after treatment. Similarly, for the TS491 tea clone, ITS1–ITS4 represent the respective treatments before application, while FTS1–FTS4 correspond to the samples after treatment. Detailed treatment descriptions are provided in the main text.

The functional annotation of metagenomic data using KEGG pathways reveals significant alterations in the microbial functional profiles across the treatments involving the chemical fertilizer and bacterial consortium application in the two tea clones, S3A3 and TS491. Notably, the consortium-treated soils exhibited a distinct enrichment in genes associated with key plant growth-promoting functions compared to the fertilizer-treated soils, emphasizing the role of rhizosphere microbiota in sustainable nutrient management. Heatmap clustering demonstrates the clear segregation of the microbial community functions between the fertilizer- and consortium-treated soils. Additionally, functional genes related to ABC transporters, amino sugar and nucleotide sugar metabolism, glycolysis, amino acid metabolism, and the biosynthesis of membrane macrolides were elevated in the consortium-treated samples compared to the controls. The KEGG level 1 analysis revealed significant changes in the gene abundance, affecting major protein groups, including metabolism, environmental information processing, genetic information processing, and cellular functions (Figure 6). Such metabolic richness likely supports improved nutrient acquisition, signaling, and defense priming in tea plants [93]. The KEGG level 3 analysis indicated that the treatments with the bacterial consortium and fertilizer bio-stimulation increased the functional abundances in the plant varieties S3A3 and TS491. In the present study, with the consortium treatment (both S3A3 and TS491), notable enhancements were observed in several metabolic pathways of the rhizosphere, including indole alkaloid biosynthesis, glutathione metabolism, nitrogen metabolism, and non-ribosomal peptide siderophore biosynthesis. The examination of the metabolic pathways revealed that the modifications among the rhizosphere soils treated with the various fertilizers were primarily caused by the enhanced pathways linked to the metabolism of sugars, amino acids, and alkaloids. It is well known that macrolides suppress a variety of diseases, including those that are caused by pathogenic fungi [94]. The KEGG analysis revealed the elevated expressions of genes involved in indole alkaloid biosynthesis and other secondary metabolite pathways (Figure 7A,B) in the consortium-treated groups. These compounds are not only plant defense molecules but may also modulate plant–microbe interactions. This observation suggests that the bacterial consortium fosters a microbiome capable of supporting systemic resistance in tea plants. Genes involved in glutathione metabolism, a crucial antioxidant defense pathway, were significantly enriched in the consortium-treated soils (Figure 7C,D). This suggests that microbial consortia may contribute to redox homeostasis in the rhizosphere, enhancing the tea plant tolerance to abiotic stresses such as drought or oxidative stress [95]. Notably, the sharp decline in glutathione metabolism genes in the ISS2 and ISS3 samples suggests that chemical fertilizers may suppress microbial functions related to stress alleviation.

The metagenomic sequences related to nitrogen metabolism were markedly elevated in the consortium-treated samples (ISS2–FSS2, ITS2–FTS2) relative to those treated solely with chemical fertilizers (ISS3–FSS3, ITS3–FTS3), with both tea clones, S3A3 and TS491, showing particularly pronounced enhancements (Figure 7E,F). This indicates that the bacterial consortium likely promotes nitrogen cycling through the enrichment of genes involved in nitrification, ammonification, and possibly nitrogen fixation. Such shifts suggest that the consortium facilitates a more biologically driven nitrogen supply to tea plants, potentially reducing the reliance on synthetic nitrogen inputs. The consortium-treated soils also showed higher abundances of genes linked to the non-ribosomal peptide synthesis of siderophores, which are critical for iron acquisition in iron-limited soils (Figure 7G,H). These microbial metabolites can improve plant iron nutrition while suppressing pathogens by competing for iron, aligning with previous findings on the biocontrol potential of PGPRs in tea. In contrast, a notable reduction in siderophore biosynthesis was observed in ISS2, further demonstrating the suppressive effect of chemical fertilizers on beneficial microbial functions. The difference in the functional profiles between the two clones was evident. Clone TS491 (FTS series) showed more consistent enrichment in multiple PGP-related pathways upon consortium treatment compared to clone S3A3 (FS series). This may be due to the inherent genotypic differences influencing root exudation and microbial recruitment, as previously reported in plant–microbe interaction studies. The findings of this experiment indicated the applicability of microbial inoculants in reducing the load of inorganic fertilizers in tea plants. To lower the cost and carbon footprint of producing tea, Barooah suggested the use of organic manure and microbial biofertilizers as crucial elements of the Integrated Nutrient Management (INM) schedule, which is necessary for tea planters to transition to soil health and sustained productivity [96]. This study has demonstrated that the application of a bacterial consortium enhances plant health by improving enzymatic responses, particularly in antioxidative enzymes, and improves the plant stress tolerance and overall physiological performance, thereby modulating the soil microbial community more effectively than synthetic fertilizers.

## 4. Conclusions

This study demonstrated the potential of a plant growth-promoting bacterial (PGPB) consortium as a sustainable alternative to conventional NPK fertilizers in tea cultivation. The bacterial consortium not only improved key agronomic parameters such as the shoot length, leaf area, chlorophyll content, and nutrient availability but also contributed to enhanced microbial diversity and soil enzyme activities. PGPB significantly enhanced the tea plant growth and soil health compared to conventional chemical fertilizers. These findings highlight the superior reliability and long-term benefits of using PGPB as a sustainable alternative to chemical inputs. PGPB-based biofertilization shows promise as a sustainable and climate-resilient tea production method by promoting climate action (SDG 13), food security (SDG 2), responsible consumption (SDG 12), and biodiversity conservation (SDG 15). The use of synthetic fertilizers in tea gardens can be significantly reduced by utilizing microbial formulations, such as biofertilizers, that incorporate these rhizobacterial species. Future studies must, however, investigate the interaction of these rhizobacterial isolates with other elements of the tea ecosystem. In addition to protecting plants from biotic and abiotic stresses, the present microbial consortium inoculations, linked to natural microbiota with multiple plant-beneficial functions, could be utilized to enhance tea growth, yields, and quality.

## Figures and Tables

**Table 1:** Plant growth-promoting attributes of the rhizobacterial isolates of tea.

Isolates	Phosphate Solubilization (µg/mL)	Siderophores (%)	IAA (µg/mL)	ACC Deaminase Activity	Ammonia Production *	Zinc Solubilization *	HCN Production *
**BS14**	49.45 ± 0.11	49.31 ± 0.49	90.62 ± 0.21	100.05 ± 0.03	+	−	−
**CIC2**	57.09 ± 0.11	41.77 ± 0.45	182.12 ± 0.10	85.96 ± 0.03	+	+	+
**FS1**	44.07 ± 0.10	63.84 ± 0.09	121.49 ± 0.10	89.81 ± 0.03	+	−	+
**PH3**	25.37 ± 0.10	43.59 ± 0.07	39.10 ± 0.10	100.05 ± 0.03	+	−	+
**SS2**	23.95 ± 0.10	65.73 ± 0.45	16.94 ± 0.07	80.32 ± 0.33	+	−	−
**SRH1**	34.76 ± 0.09	41.27 ± 0.19	25.60 ± 0.10	85.31 ± 0.00	+	−	−
**WJB5**	57.77 ± 0.11	62.54 ± 0.17	36.55 ± 0.14	62.82 ± 0.03	+	−	−
**WJB6**	35.30 ± 0.00	66.91 ± 0.13	15.99 ± 0.14	62.82 ± 0.03	+	+	+

Values are averages of three replicates ± SDs; * estimated qualitatively; (+) positive and (−) negative results for the test.

**Table 2:** Effects of the treatments on (**A**) growth parameters and (**B**) chlorophyll contents of tea clones (S3A3 and TS491) under different treatments during the 12-month field trial.

(A) Growth Parameters		S3A3				TS491			
Parameters	Treatment/Duration (Months)	Control	Consortium	50% Fertilizer + Consortium	Only Fertilizer	Control	Consortium	50% Fertilizer + Consortium	Only Fertilizer
**Shoot Length**	**0**	15.6 ± 0.14 ^a^	15.7 ± 0.29 ^a^	15.4 ± 0.36 ^a^	15.6 ± 0.29 ^a^	14.8 ± 0.24 ^a^	15.1 ± 0.24 ^a^	14.8 ± 0.24 ^a^	15.5 ± 0.76 ^a^
	**12**	59.1 ± 4.49 ^b^	86.2 ± 0.90 ^d^	68.1 ± 0.29 ^c^	48.13 ± 0.6 ^a^	56.6 ± 0.43 ^b^	88.5 ± 0.42 ^c^	59.8 ± 4.84 ^b^	46.1 ± 1.51 ^a^
**Root Length**	**0**	5.1 ± 0.662 ^a^	5.5 ± 0.81 ^a^	5.5 ± 0.36 ^a^	4.2 ± 0.59 ^a^	4.8 ± 0.34 ^b^	4.7 ± 0.17 ^a^	4.9 ± 0.45 ^a^	4.8 ± 0.26 ^a^
	**12**	10.7 ± 0.95 ^a^	14.1 ± 0.66 ^b^	10.8 ± 1.03 ^a^	9.6 ± 0.98 ^a^	11.4 ± 1.84 ^a^	13 ± 0.82 ^b^	11.6 ± 1.25 ^a^	11.3 ± 1.69 ^a^
**Leaf Number**	**0**	7.5 ± 0.47 ^a^	7.5 ± 0.47 ^a^	6.0 ± 0.47 ^a^	6.5 ± 0.82 ^a^	7.0 ± 0.82 ^a^	7.6 ± 0.47 ^a^	6.3 ± 0.47 ^a^	7.0 ± 0.82 ^a^
	**12**	28.3 ± 2.05 ^a^	96.3 ± 6.79 ^c^	73.6 ± 3.3 ^b^	44.0 ± 6.16 ^a^	30.6 ± 2.05 ^a^	99.6 ± 3.86 ^c^	58.0 ± 5.72 ^b^	36.3 ± 6.9 ^a^
**Water Content**	**12**	55.4 ± 0.44 ^a^	78.7 ± 0.50 ^d^	69.5 ± 1.35 ^c^	61.8 ± 0.80 ^b^	54.6 ± 0.57 ^b^	76.2 ± 0.23 ^d^	69.1 ± 0.43 ^c^	60.32 ± 0.37 ^a^
**(B) Chlorophyll Content**	**S3 A3**					**TS491**			
**Duration (Months)**	**Treatment/** **Chlorophyll Content (µg/g)**	**Control**	**Consortium**	**50% Fertilizer + Consortium**	**Only Fertilizer**	**Control**	**Consortium**	**50% Fertilizer + Consortium**	**Only Fertilizer**
**3 months**	Chl a	1.80 ± 0.00 ^a^	5.60 ± 0.00 ^d^	4.90 ± 0.00 ^c^	4.30 ± 0.00 ^b^	2.20 ± 0.00 ^a^	6.10 ± 0.00 ^d^	5.30 ± 5.81 ^c^	4.90 ± 0.00 ^b^
	Chl b	2.80 ± 0.00 ^a^	5.00 ± 0.00 ^c^	3.40 ± 0.00 ^b^	2.20 ± 0.00 ^a^	6.30 ± 0.00 ^a^	5.40 ± 0.00 ^d^	5.50 ± 0.00 ^c^	2.60 ± 0.00 ^b^
	Carotene	1.91 ± 0.00 ^a^	3.20 ± 0.01 ^d^	2.84 ± 0.00 ^c^	2.83 ± 0.00 ^b^	1.23 ± 0.07 ^a^	3.19 ± 0.20 ^d^	2.81 ± 0.05 ^c^	2.32 ± 0.04 ^b^
**6 months**	Chl a	2.60 ± 0.00 ^a^	8.00 ± 0.00 ^d^	7.20 ± 0.00 ^c^	5.30 ± 0.00 ^b^	3.10 ± 0.01 ^a^	8.20 ± 0.03 ^d^	8.10 ± 0.02 ^c^	2.60 ± 0.02 ^b^
	Chl b	2.00 ± 0.00 ^c^	1.40 ± 0.00 ^b^	1.40 ± 0.00 ^b^	1.10 ± 0.00 ^a^	1.20 ± 0.01 ^a^	6.40 ± 0.01 ^c^	2.00 ± 0.01 ^b^	2.40 ± 0.01 ^b^
	Carotene	3.43 ± 0.02 ^c^	1.86 ± 0.18 ^a^	2.67 ± 0.00 ^b^	1.71 ± 0.01 ^a^	1.73 ± 0.11 ^a^	3.22 ± 0.21 ^c^	2.43 ± 0.041 ^b^	1.28 ± 0.00 ^a^
**9 months**	Chl a	2.70 ± 0.00 ^a^	7.50 ± 0.00 ^d^	7.00 ± 0.00 ^c^	5.80 ± 0.00 ^b^	3.10 ± 0.00 ^a^	8.10 ± 0.00 ^c^	8.70 ± 0.00 ^c^	6.60 ± 0.00 ^b^
	Chl b	5.40 ± 0.00 ^b^	8.20 ± 0.00 ^c^	4.20 ± 0.00 ^b^	2.50 ± 0.00 ^a^	5.60 ± 0.00 ^b^	6.10 ± 0.00 ^c^	2.00 ± 0.00 ^a^	2.20 ± 0.00 ^a^
	Carotene	2.17 ± 0.00 ^a^	3.39 ± 0.00 ^d^	3.13 ± 0.00 ^c^	3.03 ± 0.00 ^b^	1.74 ± 0.018 ^a^	4.02 ± 0.00 ^c^	3.78 ± 0.39 ^c^	3.00 ± 0.00 ^b^
**12 months**	Chl a	3.10 ± 0.00 ^a^	7.60 ± 0.00 ^d^	6.30 ± 0.00 ^c^	5.60 ± 0.00 ^b^	4.60 ± 0.00 ^a^	11.00 ± 0.00 ^d^	9.4 ± 0.00 ^c^	8.50 ± 0.00 ^b^
	Chl b	5.900 ± 0.00 ^a^	12.00 ± 6.00 ^d^	11.00 ± 0.00 ^c^	10.00 ± 0.00 ^b^	6.20 ± 0.00 ^c^	11.00 ± 0.00 ^d^	7.00 ± 0.00 ^a^	3.70 ± 0.00 ^b^
	Carotene	2.42 ± 0.02 ^a^	3.80 ± 0.01 ^d^	3.28 ± 0.01 ^c^	2.92 ± 0.00 ^b^	2.12 ± 0.00 ^a^	4.72 ± 0.01 ^d^	4.45 ± 0.0 ^c^	3.57 ± 0.00 ^b^

Data are means ± SDs of triplicate samples. Chl a: Chlorophyll a; Chl b: Chlorophyll b. Values represented by distinct alphabets exhibited statistically significant variations (*p* < 0.05), whereas identical alphabets revealed no significant variances.

## Data Availability

The 16S rRNA sequences were deposited to the NCBI and obtained the NCBI GenBank accession numbers OQ799287.1, OQ569706, OQ799921.1, OQ799288.1, OQ789244.1, OQ799289.1, OQ799290.1, and Q799291.1, and the raw sequencing data of the NGS-based community analysis were deposited in the sequence read archive of the NCBI under BioProject number PRJNA986307 with the following accession numbers: SRR25000503 ISS1; SRR25000490 FSS1; SRR25000502 ISS2; SRR25000495 FSS2; SRR25000488 ISS3; SRR25000494 FSS3; SRR25000489 ISS4; SRR25000493 FSS4; SRR25000492 ITS1; SRR25000499 FTS1; SRR25000491 ITS2; SRR25000498 FTS2; SRR25000501 ITS3; SRR25000497 FTS3; SRR25000500 ITS4; SRR25000496 FTS4.

## Supplementary Materials

The following supporting information can be downloaded at: https://www.mdpi.com/article/10.3390/microorganisms13081715/s1, Figure S1: 16S rRNA gene sequence–based phylogenetic relationship of *Serratia* sp. strains FS1 (a), CIC2 (b), PH3 (c), WJB6 (d), SRH1 (e) and *Bacillus* sp. strain BS14 (f), SS2 (g), WJB5 (h) with its closest respective bacterial strains. *Bradyrhizobium japonicum* USDA 6 used as the out-group. The sequences were aligned by Muscle and analyzed by neighbor joining (Kimura 2-parameter model). Bootstrap values based on 1000 replicates are listed as percentages at the nodes; Figure S2: Experimental pot trial setup under controlled greenhouse conditions for *C. sinensis*, assessing the response of two tea cultivars, (A) S3A3 and (B) TS491, to varying treatment conditions; Figure S3: Evaluation of enzyme activity of Pot trial experiment to show the effects with different treatments in two tea clones S3A3 and TS491: Phenolic content (A,B), CAT activity (C,D), glutathione reductase (E, F) and SOD activity (G,H). Values marked with various symbols (*, **, ***) indicate significant variations (*p* ≤ 0.05); Figure S4: Effect of treatments on soil enzyme activities as in Pot trial—FDA (A,B) and DHA (C,D) of tea clone S3A3 and TS491. Values marked with various symbols (*, **, ***) indicate significant variations (*p* ≤ 0.05); Figure S5: Effect of Rhizobacteria treatments on soil enzyme activities as in field trial with tea variety S3A3 and TS491.Fluorescein diacetate (FDA) activity (I.a,II.a) and Dehydrogenase (DHA) activity (I.b,II.b). Values marked with various symbols (*, **, ***) indicate significant variations (*p* ≤ 0.05); Table S1: Effect of bacterial consortium on tea plants (A) plant growth parameters and (B) chlorophyll content in Pot trials; Table S2: Determination of soil characteristics before and after treatments.

## Abbreviations

ISS1	Initial S3A3 Sample 1 (control, before treatment)
ISS2	Initial S3A3 Sample 2 (consortium, before treatment)
ISS3	Initial S3A3 Sample 3 (50% fertilizer + consortium, before treatment)
ISS4	Initial S3A3 Sample 4 (fertilizer only, before treatment)
FSS1	Final S3A3 Sample 1 (control, after treatment)
FSS2	Final S3A3 Sample 2 (consortium, after treatment)
FSS3	Final S3A3 Sample 3 (50% fertilizer + consortium, after treatment)
FSS4	Final S3A3 Sample 4 (fertilizer only, after treatment)
FTS1	Final TS491 Sample 1 (control, after treatment)
FTS2	Final TS491 Sample 2 (consortium, after treatment)
FTS3	Final TS491 Sample 3 (50% fertilizer + consortium, after treatment)
FTS4	Final TS491 Sample 4 (fertilizer only, after treatment)
ITS1	Initial TS491 Sample 1 (control, before treatment)
ITS2	Initial TS491 Sample 2 (consortium, before treatment)
ITS3	Initial TS491 Sample 3 (50% fertilizer + consortium, before treatment)
ITS4	Initial TS491 Sample 4 (fertilizer only, before treatment)
