# Supplementary material for: Sustainable Tea Cultivation with a Rhizobacterial Consortium: A Microbiome-Driven Alternative to Chemical Fertilizers
Source: Microorganisms. 2025 Jul 22;13(8):1715. doi: 10.3390/microorganisms13081715 (PMC12388101; doi:10.3390/microorganisms13081715)
Supplement: Supplementary file 1 [file microorganisms-13-01715-s001.zip › microorganisms-3717076-supplementary.pdf]

Supplementary Data

S1. Results of 16S rRNA gene sequence–based phylogenetic relationship:

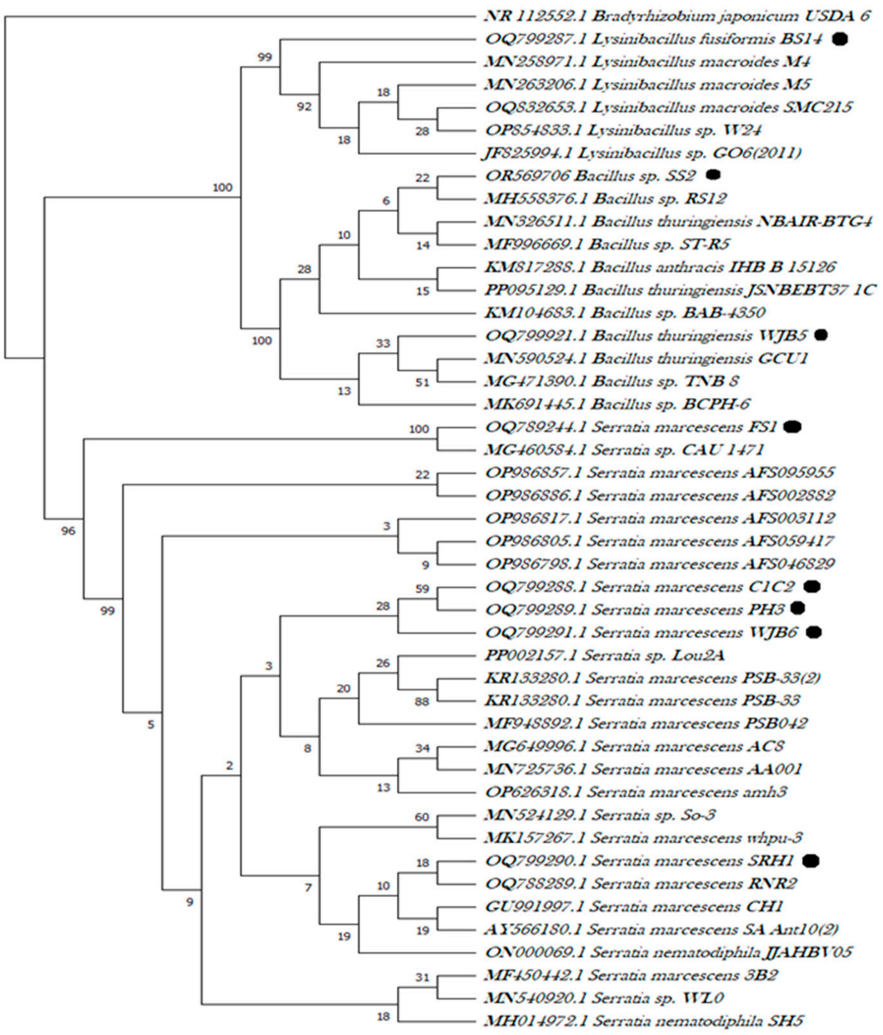

Figure S1. 16S rRNA gene sequence–based phylogenetic relationship of *Serratia* sp. strains FS1 (a), CIC2 (b), PH3 (c), WJB6 (d), SRH1 (e) and *Bacillus* sp. strain BS14 (f), SS2 (g), WJB5 (h) with its closest respective bacterial strains. *Bradyrhizobium japonicum* USDA 6 used as the out-group. The sequences were aligned by Muscle and analyzed by neighbor joining (Kimura 2-parameter model). Bootstrap values based on 1000 replicates are listed as percentages at the nodes.

S2. Results of pot trials assay with the plants *Camellia sinensis* (S3A3 and TS491) clones

S2.1. Growth and Physiological Improvements in Tea plants under bacterial inoculation:

The pot trial set up under greenhouse with different treatments are shown in **Figure S2 (A and B)**. After 90 and 180 days, both clones exhibited increased height and leaf numbers compared to the uninoculated control. In the bacterial inoculated tea plant the shoot length was found to increase by 19% in tea clone S3A3 and in tea clone TS491 it was recorded to be increased by 37% whereas in 50% amended fertilizer it was found to be 17% and 25% (S3A3 and TS491) respectively. Leaf numbers in both the tea clones were increased by 9-11% after inoculation of the consortium. Water content and chlorophyll levels were also maximized in consortium-treated plants, with chlorophyll a at 19.00 mg/g (S3A3) and 13.00 mg/g (TS491), Chlorophyll b 18.00 mg/g (S3A3) and 13.00 mg/g (TS491) and carotene content at 4.95mg/g (S3A3) and 7.87 mg/g (TS491) respectively.

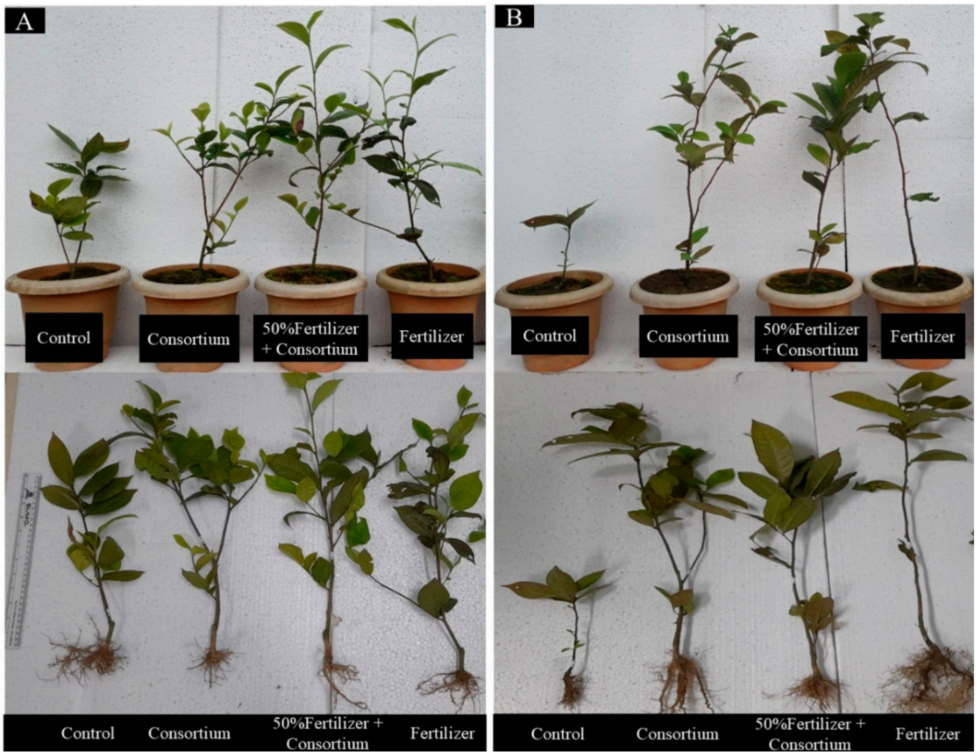

Figure S2. Experimental pot trial setup under controlled greenhouse conditions for C. *sinensis*, assessing the response of two tea cultivars, (A) S3A3 and (B) TS491, to varying treatment conditions.

S2.2. Effect of rhizobacteria on the plant enzyme during pot trial assay:

S2.2.1. Phenol content: In both tea clones (S3A3 and TS491), plants treated with the bacterial consortium exhibited the highest accumulation of phenolics, compared to the first harvest at 90 days, with S3A3 showing values of 1050.04 to 1543.39 mg GAE/ml and TS491 ranging from 951.82 to 1616.49 mg GAE/ml which suggesting enhanced secondary metabolism. The 50% Fertilizer + Consortium treatment (S3A3:1348.93 mg GAE/ml; TS491:1243.82 mg GAE/ml) also resulted in increased phenolic content compared to control and only fertilizer, though slightly lower than the consortium-only treatment. The only fertilizer treatment showed no significant increase in phenolic content over the control **Figure S3 (A and B).**

S2.2.2. Catalase (CAT) activity: Harvests at 90 and 180 days demonstrated increased catalase activity in all treated plants, with the highest levels observed in tea plants amended with a bacterial consortium for both clones S3A3 and TS491. Catalase concentrations were slightly different between the clones (S3A3: 377.84 to 500.37 U/g/min; TS491: 466.95 to 499.97 U/g/min). The 50% Fertilizer + Consortium treatment also exhibited higher CAT activity than control and only fertilizer **Figure S3 (C and D).**

S2.2.3. Glutathione reductase (GR) activity: Glutathione reductase (GR) is a key enzyme in the plant antioxidative system. The highest glutathione levels were observed in bacterial-treated plants for both tea clones, with S3A3 at 482.44 µmole/gfw and TS491 at 478.72 µmole/gfw. This was followed by plants amended with 50% fertilizer and a bacterial consortium, showing S3A3 at 391.89 µmole/gfw and TS491 at 370.33 µmole/gfw shown in **Figure S3 (E and F).**

S2.2.4. Superoxide dismutase (SOD) activity: In this study, after 180 days of treatments both the consortium-augmented plants and those receiving fertilizer with the consortium exhibited markedly higher SOD activity, with S3A3 showing 415.71 unit/gfw/sec and TS491 at 418.71 unit/gfw/sec, compared to other treatments **Figure S3 (G and H**).

S2.3. Effect of Bacterial Consortium on Soil Enzyme Activities (FDA and DHA) in Tea under greenhouse condition

S2.3.1. Fluorescein diacetate (FDA) activity: The soil FDA hydrolysis activity was assessed for all the four treatments with *C. sinensis* S3A3, the addition of a bacterial consortium significantly enhanced FDA hydrolase activity compared to both the control and fertilizer-only treatments after 180 days (S3A3: 9.66 mg fluorescein/g soil; TS491: 5.67 mg fluorescein/g soil). A similar trend was observed in *C. sinensis* TS491, where consortium-treated plants exhibited higher FDA hydrolase activity **Figure S4 (A and B**).

S2.3.2. Dehydrogenase (DHA) activity: For the pot trial experiment with *C. sinensis* S3A3, the dehydrogenase activity was found to increase with the amendment of bacterial consortium as compared to fertilizer amended plants and control after a period of 180 days was 0.066 formazan mg/g soil in S3A3. The same trend was followed in *C. sinensis* TS491, where the dehydrogenase activity was highest for the consortium amended soil followed by consortium+50%fertilizer amended soil was 0.065 formazan mg/g soil in TS491 **Figure S4 (C and D**).

Table S1: Effect of bacterial consortium on tea plants (A) plant growth parameters and (B) chlorophyll content in Pot trials.

A Tea clone		S3A3				TS491			
**Parameters**	**Treatment/Duration (Months)**	**Control**	**Consortium**	**50% fertilizer + consortium**	**Only fertilizer**	**Control**	**Consortium**	**50% fertilizer + consortium**	**Only fertilizer**
**Shoot length**	**0**	15.60 ± 0.14^{a}	15.70 ± 0.29^{a}	15.37 ± 0.37^{a}	15.60 ± 0.29^{a}	14.83 ± 0.24^{a}	15.17 ± 0.24^{a}	14.83 ± 0.24^{a}	15.57 ± 0.76^{a}
	**6**	30.67 ± 0.47^{a}	49.53 ± 0.41^{c}	47.93 ± 0.^{d}	36.33 ± 0.24^{b}	19.50 ± 0.41^{a}	56.30 ± 0.50^{c}	45.33 ± 0.12^{c}	27.40 ± 0.49^{b}
**Root length**	**0**	4.87 ± 0.63^{a}	4.70 ± 0.22^{a}	4.30 ± 0.62^{a}	5.00 ± 0.82^{a}	3.83 ± 0.24^{a}	4.47 ± 0.45^{a}	5.22 ± 2.43^{a}	5.35 ± 1.95^{a}
	**6**	12.23 ± 0.2^{b}	22.83 ± 1.43^{c}	9.93 ± 0.48^{b}	7.23 ± 0.40^{a}	7.07 ± 0.25^{a}	15.60 ± 0.43^{c}	11.40 ± 0.43^{b}	7.67 ± 0.47^{a}
**Leaf Number**	**0**	5.5 ± 0.72^{a}	5.48 ± 0.74^{a}	5.38 ± 0.72^{a}	5.28 ± 0.65^{a}	5.4 ± 0.61^{a}	5.33 ± 0.62^{a}	5.16 ± 0.68^{a}	5.33 ± 0.47^{a}
	**6**	21.9 ± 10.60^{a}	22.96 ± 10.65^{c}	20.47 ± 9.56^{b}	19.22 ± 9.73^{b}	18.73 ± 10.57^{a}	21.75 ± 9.69^{c}	15.66 ± 2.69^{b}	14 ± 0.82^{b}
**Water Content**	**6**	49.10 ± 0.59^{a}	56.91 ± 3.23^{c}	50.87 ± 1.12^{b}	51.41 ± 0.82^{b}	47.79 ± 1.89^{a}	52.83 ± 1.98^{c}	47.25 ± 3.10^{a}	50.37 ± 1.42^{b}
**B Tea clone**		**S3A3**				**TS491**			
**Duration**	**Treatment Chlorophyll content (µg/g)**	**Control**	**Consortium**	**50% fertilizer + consortium**	**Only fertilizer**	**Control**	**Consortium**	**50% fertilizer + consortium**	**Only fertilizer**
**3**	Chl a	5.90 ± 0.00^{a}	9.30 ± 0.00^{d}	7.20 ± 0.00^{c}	6.30 ± 0.00^{b}	3.00 ± 0.00^{a}	7.00 ± 0.00^{d}	6.00 ± 0.00^{c}	5.00 ± 0.00^{b}
	Chl b	6.60 ± 0.00^{a}	8.90 ± 0.00^{c}	6.60 ± 0.00^{b}	6.80± 0.00^{b}	2.00 ± 0.00^{a}	5.00 ± 0.00^{d}	2.00 ± 0.00^{c}	1.00 ± 0.00^{b}
	Carotene	3.30 ± 0.04^{a}	3.98 ± 0.12^{c}	3.74 ± 0.04^{b}	3.23 ± 0.035^{a}	1.69 ± 0.06^{a}	3.74 ± 0.00^{d}	3.051 ± 0.07^{c}	2.15 ± 0.20^{b}
**6**	Chl a	12.00 ± 0.00^{a}	0.019 ± 0.00^{c}	13.00 ± 0.00^{b}	12.01 ± 0.00^{a}	10.00 ± 0.00^{a}	13.00 ± 0.00^{b}	11.00 ± 0.00^{a}	11.00 ± 0.00^{a}
	Chl b	11.00 ± 0.00^{a}	18.00 ± 0.00^{c}	12.00 ± 0.00^{b}	9.00 ± 0.00^{ab}	6.00 ± 0.00^{a}	11.00 ± 0.00^{d}	8.00 ± 0.00^{c}	7.00 ± 0.00^{b}
	Carotene	3.28 ± 0.01^{a}	4.96 ± 0.02^{d}	4.00 ± 0.01^{c}	3.69 ± 0.00^{b}	4.64 ± 0.03^{a}	7.87 ± 0.19^{d}	7.27 ± 0.028^{c}	5.97 ± 0.01^{b}

Data are mean ± SD of triplicate samples. Chl a = Chlorophyll a; Chl b = Chlorophyll b.Values represent mean±SE. Values represented by distinct alphabets exhibited statistically significant variations ($p < 0.05$), whereas identical alphabets revealed no significant variances

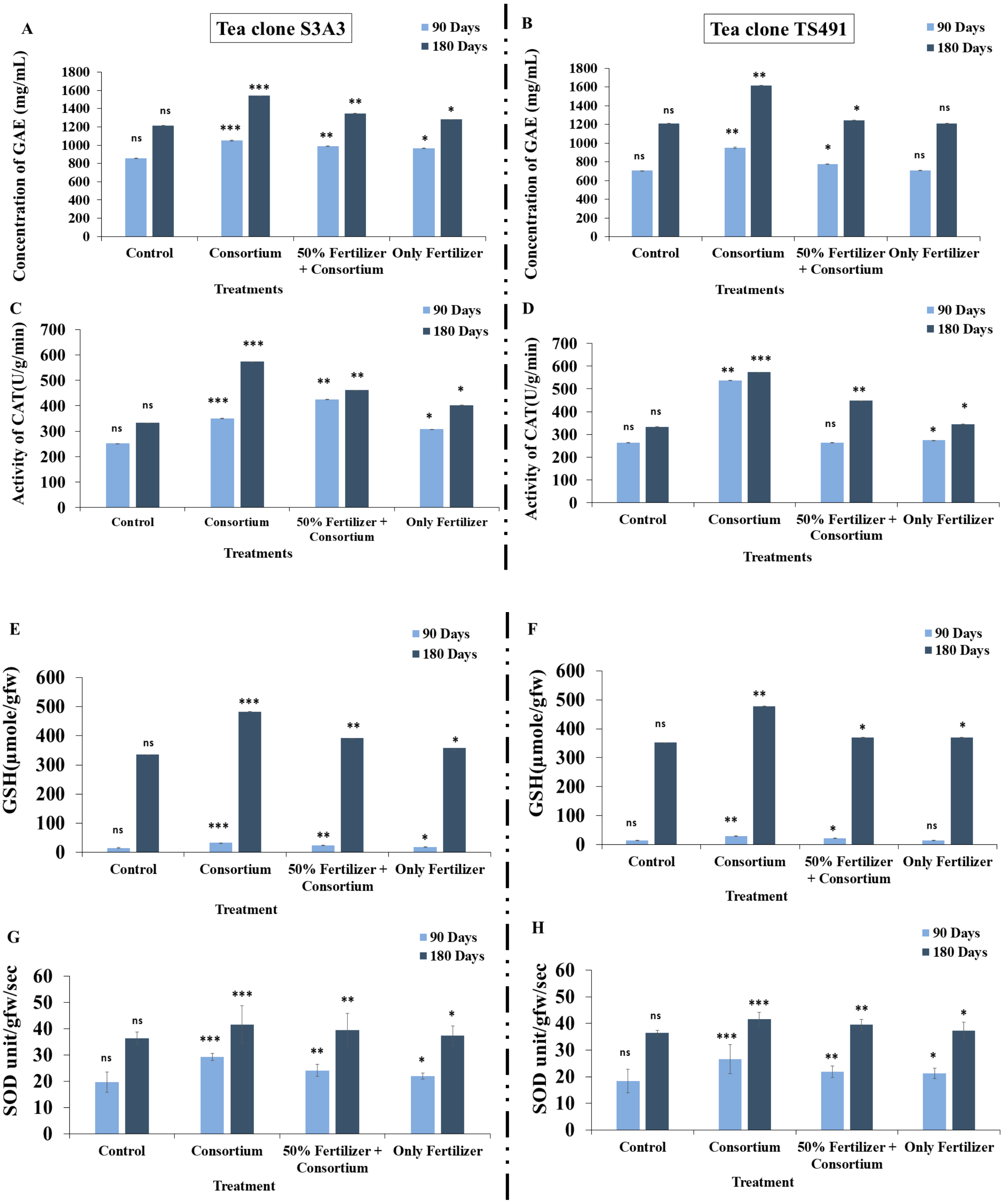

Figure S3. Evaluation of enzyme activity of Pot trial experiment to show the effects with different treatments in two tea clones S3A3 and TS491: Phenolic content (A and B), CAT activity (C and D), glutathione reductase (E and F) and SOD activity (G and H). Values marked with various symbols (*, **, ***) indicate significant variations ($P \leq 0.05$)

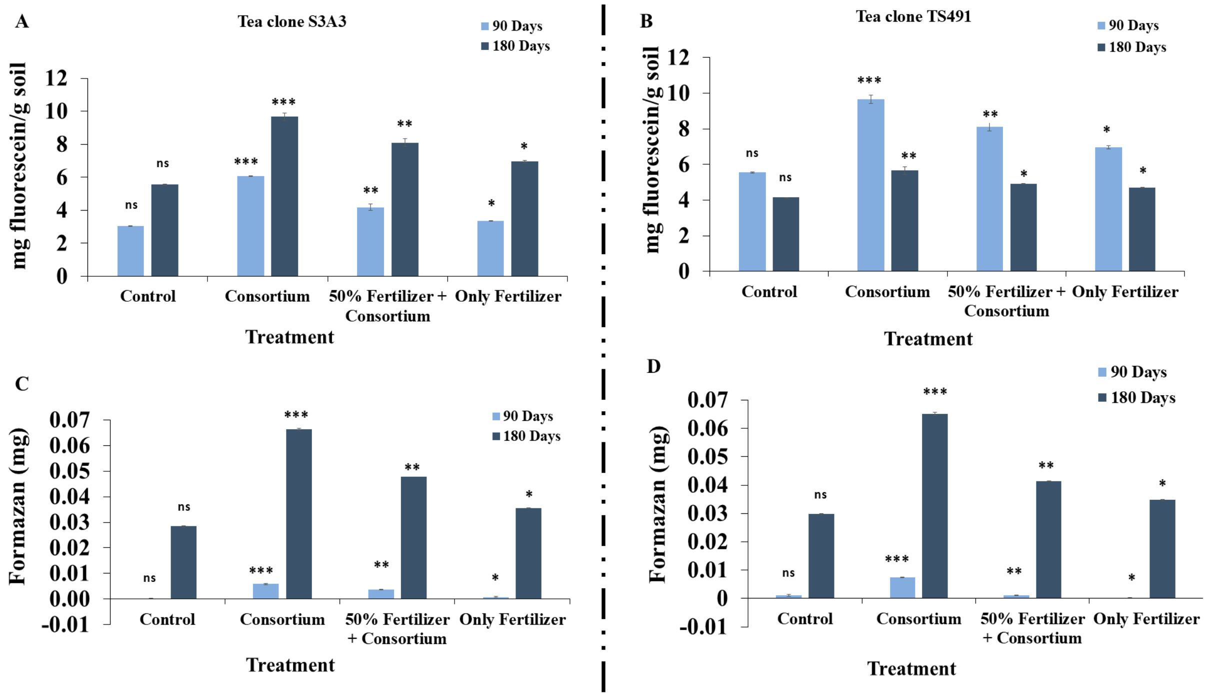

Figure S4. Effect of treatments on soil enzyme activities as in Pot trial- FDA (A and B) and DHA (C and D) of tea clone S3A3 and TS491. Values marked with various symbols (*, **, ***) indicate significant variations (P≤ 0.05)

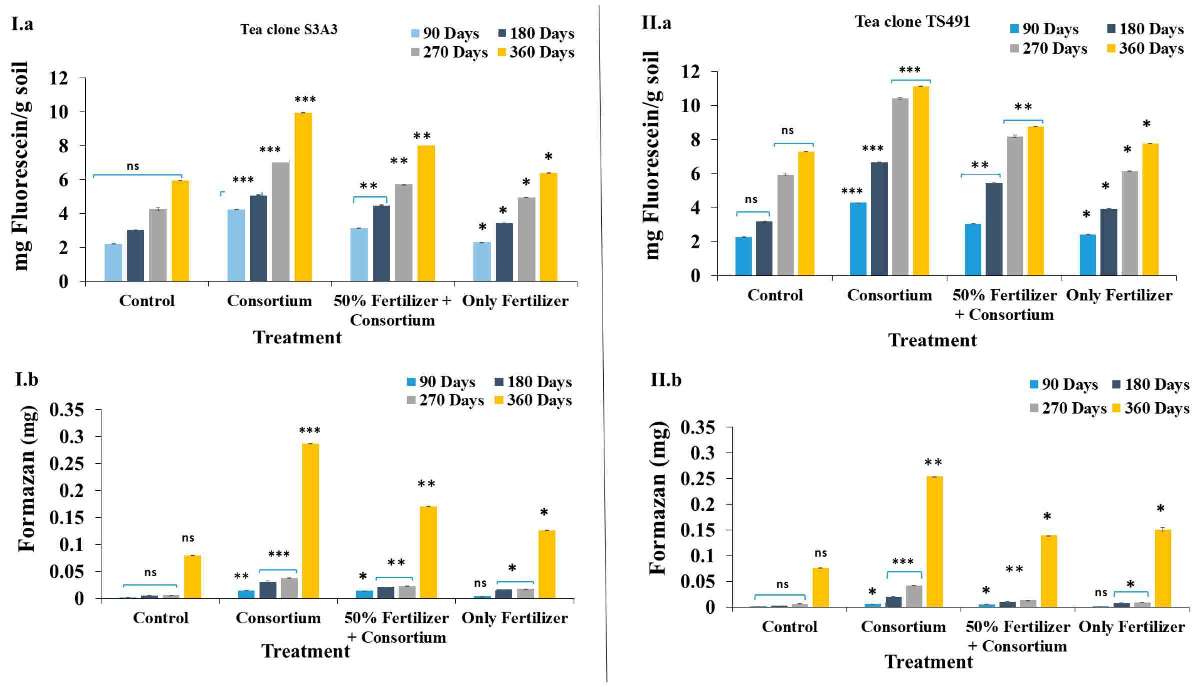

Figure S5. Effect of Rhizobacteria treatments on soil enzyme activities as in field trial with tea variety S3A3 and TS491.Fluorescein diacetate (FDA) activity (I.a and II.a) and Dehydrogenase (DHA) activity (I.b and II.b). Values marked with various symbols (*, **, ***) indicate significant variations. . * $p \leq 0.05$ (significant), ** $p \leq 0.01$ (highly significant), *** $p \leq 0.001$ (very highly significant).

Table S2: Determination of soil characteristics before and after treatments.

Soil Characteristic	Before treatment	After treatment							
		S3A3				TS491			
	Bulk soil	Control	Bacterial Consortium	50% Fertilizer + Consortium	Only Fertilizer	Control	Bacterial Consortium	50% Fertilizer + Consortium	Only Fertilizer
pH	3.90 ± 0.01	3.86 ± 0.07^{b}	4.24 ± 0.08^{a}	3.98 ± 0.08^{b}	3.77 ± 0.08^{b}	3.86 ± 0.07^{b}	4.17 ± 0.04^{a}	3.68 ± 0.01^{b}	3.79 ± 0.03^{b}
TOC (%)	1.32 ± 0.00	1.41 ± 0.01^{b}	1.73 ± 0.09^{a}	1.51 ± 0.01^{c}	1.42 ± 0.00^{b}	1.41 ± 0.011^{b}	1.57 ±0.07^{c}	1.51 ±0.07^{c}	1.41 ±0.01^{b}
TN (mg/kg)	121.77 ±0.39	123.55± 0.69^{c}	153.34 ± 0.47^{a}	143.14 ± 0.13^{b}	141.28 ± 0.77^{b}	122.59 ± 0.41^{c}	152.34 ± 0.47^{a}	141.14 ± 0.94^{b}	140.68 ± 1.21^{b}
TP (mg/kg)	21.55 ± 0.46	22.67 ± 0.72^{b}	25.43 ± 0.28^{a}	22.99 ± 0.25^{b}	22.23 ± 0.64^{b}	22.71 ± 0.69^{b}	25.17 ± 0.02^{a}	22.86 ± 0.14^{b}	22 ± 0.15^{b}
TK (mg/kg)	20.63 ± 0.46	20.47 ± 0.46^{c}	29.59 ±0.32^{a}	27.14 ± 0.10^{b}	26.81 ±0.57^{b}	20.58 ± 0.37^{c}	29.66 ± 0.41^{a}	17.15 ± 0.09^{a}	26.96 ± 0.37^{b}

Data are mean ± SD of triplicate samples; Total organic carbon (TOC), total nitrogen (TN), total phosphorus (TP), and total potassium (TK). Values represented by distinct alphabets exhibited statistically significant variations ($p < 0.05$), whereas identical alphabets revealed no significant variances

S3. Media used, and their composition:

Pikovskaya agar: Yeast extract: 0.5 g/L, Dextrose: 10.0 g/L, Ca3$(PO_4)_2$: 5.0 g/L, $(NH_4)_2SO_4$: 0.5 g/L, KCl: 0.2 g/L, $MgSO_4$: 0.1 g/L, $MnSO_4 \cdot H_2O$: 0.0001 g/L, $FeSO_4$: 0.0001 g/L, Agar: 15.0 g/L) containing bromophenol blue. **Nutrient agar:** Peptone: 5.000 g/L, NaCl: 5.000 g/L, HM peptone B# 1.500 g/L, Yeast extract 1.500 g/L, Agar 15.000 g/L, Final pH (at 25°C) 7.4±0.2. **Nutrient Broth:** Peptone: 5.000 g/L,NaCl: 5.000 g/L, HM peptone B#: 1.500 g/L, Yeast extract: 1.500 g/L, Final pH: 7.4±0.2. **Peptone Broth:** Peptone: 10.000 g/, HM peptone B# :3.000 g/L, NaCl: 5.000 g/L, pH: 7.2±0.2. **DF (Dworkin and Foster) salts media:** KH_2PO_4: 4.0 g/L, Na_2HPO_4: 6.0 g/L, $MgSO_4$. $7H_2O$: 0.2 g/L, glucose :2.0 g/L, gluconic acid :2.0 g/L and citric acid: 2.0 g/L with trace elements: $FeSO_4$: 1 mg/L, **Mineral Salt Medium (MSM):** NaCl: 1g/L, $CaCl_2$:0.1 g/L, $MgSO_4$:0.5 g/L, KH_2PO_4 :1 g/L, K_2HPO_4 :1 g/L, yeast extract :4 g/L, agar :16–18 g/L, pH was maintained at 7.2). Different sources of insoluble zinc salts like zinc oxide (1.244 g/L = 15.23 mM), zinc phosphate (1.3882 g/L = 4.0 mM), zinc carbonate (1.4728 g/L 11.74 mM), and zinc sulfide (1.124 g/L 11.54 mM) at final concentration of 0.1% were added in the medium individually and autoclaved at 121°C for 30 minutes**, NBRIP medium**: Glucose: 10 g/ L, [$(NH_4)_2SO_4$: 0.1 g /L, [$Ca_3(PO_4)_2$ or TCP: 5 g/L, $MgCl_2 \cdot 6H_2O$: 5 g/L, $MgSO_4$: $7H_2O$, 0.25 g/L, KCl: 0.2g/L

CAS medium preparation:

A. Blue Dye: Prepared following three solutions:

a. Solution 1: Dissolved 0.06 g of CAS (Fluka Chemicals) in 50 ml of ddH_2O.

b. Solution 2: Dissolved 0.0027 g of $FeCl_3$-6 H_2O in 10 ml of 10 mM HCl.

c. Solution 3: Dissolved 0.073 g of HDTMA in 40 ml of ddH_2O.

Mixed Solution 1 with 9 ml of Solution 2. Then mix with Solution 3. Autoclaved and stored in a plastic container/bottle.

B. Mixture solution:

a. Minimal Media 9 (MM9) Salt Solution Stock: Dissolved 15 g KH_2PO_4, 25 g NaCl, and 50 g NH_4Cl in 500 ml of ddH_2O.

b. 20% Glucose Stock: Dissolved 20 g glucose in 100 ml of ddH_2O.

c. NaOH Stock: Dissolved 25 g of NaOH in 150 ml ddH_2O; pH ~12.

d. Casamino Acid Solution: Dissolved 3 g of Casamino acid in 27 ml of ddH_2O. Extract with 3% 8-hydroxyquinoline in chloroform to remove any trace iron. The solution was filter sterilized.

C. CAS agar Preparation:

a. Added 100 ml of MM9 salt solution to 750 ml of ddH_2O.

b. Dissolved 32.24 g piperazine-N,N'-bis (2-ethanesulfonic acid) PIPES. Slowly added PIPES while stirring. While stirring, slowly brought the pH up to 6.8. (Not to exceed pH 6.8 as this will turn the solution green).

c. Added 15 g agar. Autoclaved and cooled to 50°C.

e. Added 30 ml of sterile Casamino acid solution and 10 ml of sterile 20% glucose solution to MM9/PIPES mixture.

f. Slowly added 100 ml of Blue Dye solution along the glass wall with enough agitation to mix thoroughly.

g. Aseptically poured in petri plates.
